# The *Bifidobacterium dentium* Bd1 Genome Sequence Reflects Its Genetic Adaptation to the Human Oral Cavity

**DOI:** 10.1371/journal.pgen.1000785

**Published:** 2009-12-24

**Authors:** Marco Ventura, Francesca Turroni, Aldert Zomer, Elena Foroni, Vanessa Giubellini, Francesca Bottacini, Carlos Canchaya, Marcus J. Claesson, Fei He, Maria Mantzourani, Laura Mulas, Alberto Ferrarini, Beile Gao, Massimo Delledonne, Bernard Henrissat, Pedro Coutinho, Marco Oggioni, Radhey S. Gupta, Ziding Zhang, David Beighton, Gerald F. Fitzgerald, Paul W. O'Toole, Douwe van Sinderen

**Affiliations:** 1 Laboratory of Probiogenomics, Department of Genetics, Biology of Microorganisms, Anthropology, and Evolution, University of Parma, Parma, Italy; 2 Alimentary Pharmabiotic Centre and Department of Microbiology, Bioscience Institute, National University of Ireland, Cork, Ireland; 3 College of Biological Sciences, China Agricultural University, Beijing, China; 4 Department of Microbiology, The Henry Wellcome Laboratories for Microbiology and Salivary Research, Kings College London Dental Institute, London, United Kingdom; 5 Department of Molecular Biology, University of Siena, Siena, Italy; 6 Dipartimento Sientifico Tecnologico, Università degli Studi di Verona, Verona, Italy; 7 Department of Biochemistry and Biomedical Sciences, McMaster University, Hamilton, Ontario, Canada; 8 Glycogenomics, Databases, and Bioinformatics, Architecture et Fonction des Macromolécules Biologiques, Universités Aix-Marseille, Marseille, France; Universidad de Sevilla, Spain

## Abstract

Bifidobacteria, one of the relatively dominant components of the human intestinal microbiota, are considered one of the key groups of beneficial intestinal bacteria (probiotic bacteria). However, in addition to health-promoting taxa, the genus *Bifidobacterium* also includes *Bifidobacterium dentium*, an opportunistic cariogenic pathogen. The genetic basis for the ability of *B. dentium* to survive in the oral cavity and contribute to caries development is not understood. The genome of *B. dentium* Bd1, a strain isolated from dental caries, was sequenced to completion to uncover a single circular 2,636,368 base pair chromosome with 2,143 predicted open reading frames. Annotation of the genome sequence revealed multiple ways in which *B. dentium* has adapted to the oral environment through specialized nutrient acquisition, defences against antimicrobials, and gene products that increase fitness and competitiveness within the oral niche. *B. dentium* Bd1 was shown to metabolize a wide variety of carbohydrates, consistent with genome-based predictions, while colonization and persistence factors implicated in tissue adhesion, acid tolerance, and the metabolism of human saliva-derived compounds were also identified. Global transcriptome analysis demonstrated that many of the genes encoding these predicted traits are highly expressed under relevant physiological conditions. This is the first report to identify, through various genomic approaches, specific genetic adaptations of a *Bifidobacterium* taxon, *Bifidobacterium dentium* Bd1, to a lifestyle as a cariogenic microorganism in the oral cavity. *In silico* analysis and comparative genomic hybridization experiments clearly reveal a high level of genome conservation among various *B. dentium* strains. The data indicate that the genome of this opportunistic cariogen has evolved through a very limited number of horizontal gene acquisition events, highlighting the narrow boundaries that separate commensals from opportunistic pathogens.

## Author Summary

The accessibility of complete bacterial genome sequences has provided important changes to the field of microbiology by significantly enhancing our understanding of the physiology, genetics, and evolutionary development of bacteria. Bifidobacteria are among such microorganisms, being mammalian commensals of biotechnological significance due to their perceived role in maintaining a balanced gastrointestinal (GIT) microflora. Bifidobacteria are therefore often applied as health-promoting or probiotic components in functional food products and represent a growing area of scientific interest. However, within the genus *Bifidobacterium* not all species provide beneficial effects on the host's health. In fact, the *Bifidobacterium dentium* species is considered an opportunistic pathogen since it has been associated with the development of dental caries. In this manuscript, we describe the complete genetic make-up of the *B. dentium* Bd1 genome and discuss functions that explain how this microorganism has adapted to the oral human cavity and imparts a cariogeneous phenotype. Moreover, we performed comparative genomic analyses of *B. dentium* genome with other bifidobacterial genomes in order to trace genetic differences/similarities between the opportunistic oral pathogen *B. dentium* Bd1 and closely related intestinal bifidobacteria.

## Introduction

Bifidobacteria are relatively abundant inhabitants of the gastrointestinal tract (GIT) of humans and animals [1]. Many bifidobacterial species, in conjunction with other members of the intestinal microbiota are believed to contribute to host nutrition, while also impacting on intestinal pH, cell proliferation and differentiation, development and activity of the immune system, and innate and acquired responses to pathogens [2]–[8]. These perceived beneficial health effects have driven commercial exploitation of bifidobacteria as live components of many functional foods and therapeutic adjuncts. However, bifidobacteria have also been isolated from the human oral cavity, where their presence is linked to the progression of tooth decay: bifidobacteria have been detected in high numbers in infected dentine from carious lesions in children [9] and have been associated with childhood dental caries [10]. *B. dentium* can be found as part of the microbiota implicated in human dental caries [10]–[16]. In recent surveys of oral bifidobacteria associated with coronal caries in adults and children [17] and root caries in adults [18], *B. dentium* was the most frequently isolated *Bifidobacterium* species, representing approximately eight percent of the culturable bacteria isolated from active carious lesions. This species is capable of acidogenesis to produce a final pH in glucose-containing media below pH 4.2 [19], sufficient to cause extensive demineralisation of tooth tissues [20]. *B. dentium* may therefore significantly contribute to the pathogenesis of dental caries which is one of the most common chronic diseases, remaining untreated in many underdeveloped countries where dental pain is often alleviated only by the loss or extraction of the affected tooth [21].

The ecological plaque hypothesis was formulated to explain the composition and phenotypic properties of the microbiota associated with caries initiation and progression [22]. This hypothesis envisages that caries is the result of environmental changes, particularly as a result of reduced intra-oral pH as a consequence of bacterial fermentation of dietary carbohydrates. When this occurs in the oral cavity, it selects for a microbiota which is more aciduric and more acidogenic than that present in the absence of caries. The environmental change results in a significant alteration in the composition of the commensal microbiota, with taxa including bifidobacteria, lactobacilli, *Actinomyces* and streptococci proliferating [11].

Complete genome sequences of relatively few human intestinal commensal bifidobacteria have been determined, being largely motivated by their perceived health-promoting activity. These include *Bifidobacterium longum* subsp. *longum* NCC2705, *B. longum* subsp. *longum* DJO10A and *B. longum* subsp. *infantis* ATCC15697, *Bifidobacterium animalis* subsp. *lactis* DSM10140 and *B. animalis* subsp. *lactis* ADO11 [23]–[27]. Here, we describe the sequence analysis of the *B. dentium* Bd1 genome. This strain was originally isolated from human dental caries [28]. Analysis of the predicted proteome together with comparisons of the genome sequence to those of intestinal bifidobacteria revealed that this bacterium has undergone specific genetic adaptations for colonization and survival in the oral cavity.

## Results

### General genome features

**Figure 1:**
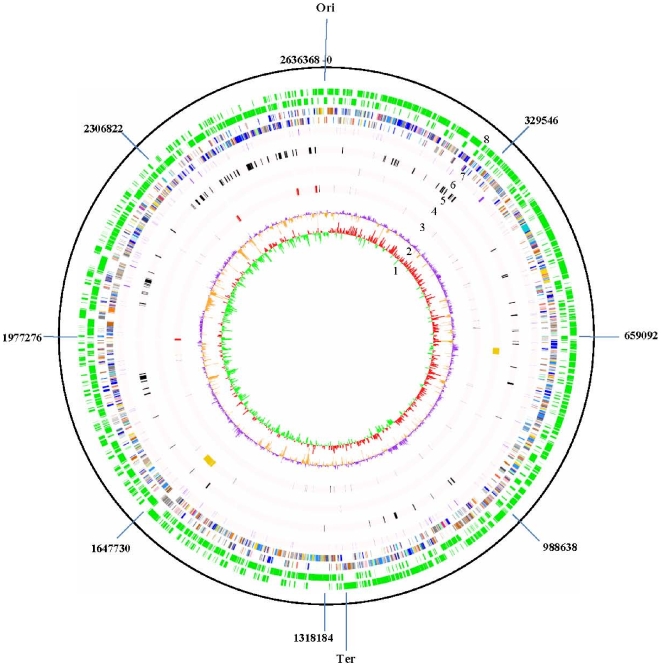
Circular genome map of *B. dentium* Bd1. From innermost circle, circle (1) illustrates GC skew (G−C/G+C), values >0 are in red and <0 in green. Circle (2) highlights G+C% deviation from the mean (58.54%). Circle (3) indicates rRNAs (depicted in red) and tRNAs (depicted in blue). Circle (4) denotes IS and prophages (depicted in orange). Circle (5) depicts genes involved in sugar metabolism according to the CAZY database. Circle (6) denotes conserved ORF distribution. Circle (7) shows coding regions by strand with color corresponding to the COG functional assignment (the color code used is the same indicated in Figure 2). Circle (8) displays the ORF distribution by strand.

The genome of *B. dentium* Bd1 is one of the largest bifidobacterial genomes reported to date, with a single circular chromosome consisting of 2,636,368 base pairs (Figure 1). The average GC content of 58.54% is similar to that of other sequenced bifidobacterial genomes and is consistent with the range of G+C mol% values for the *Actinobacteria* [1]. For protein-encoding DNA regions, the G+C contents of codon positions 1, 2 and 3 were determined to be 61%, 43%, 74%, respectively, the latter value somewhat deviating from the expected value (70%), as based on a survey of 696 eubacterial and 56 actinobacterial genomes or bifidobacterial genomes (NCBI source) (Figure S1 and data not shown).

The genome of *B. dentium* Bd1 possesses 55 tRNAs and four rRNA operons, which are located in proximity of the *ori*C. While *B. dentium* contains tRNAs for every amino acid, the corresponding genes for aminoacyl-tRNA synthetases for asparagine and glutamine appear to be absent. An alternative route is a pathway described for *Fusobacterium nucleatum* [29] that utilizes Gln-and Asn-tRNA amidotransferases, which amidate misacylated Gln-tRNA or Asn-tRNA charged with Glu or Asp to produce Gln-tRNA Gln or Asn-tRNA-Asn, respectively. Homologous genes that specify subunits for the Gln-and Asn-tRNA amidotransferase are present on the Bd1 genome and are also present on other sequenced bifidobacterial genomes (data not shown).

Identification of protein-coding sequences revealed 2143 open reading frames (ORFs) with an average length of 1059 bases and constituting 89% of the genome, the remainder representing intergenic regions with an average length of 143 bp. This latter value is lower than those calculated for other known bifidobacterial genomes, whose combined average intergenic region length is 191 bp, indicating that *B. dentium* Bd1 has a more compact genome. Such results are not biased due the methods used for *B. dentium* Bd1 genome annotation (see Text S1), since it employed an ORF identification protocol, with cut-off values that are similar to those used for the annotation of the so far published bifidobacterial genome sequences [23]–[27]. The ORFs are organised in a typical bacterial configuration, so that transcription is frequently in the same direction as DNA replication. A functional assignment was made for 78.5% of the predicted ORFs, while homologs with no known function from other bacterial species were identified for an additional 13% of the *B. dentium* Bd1 ORFs. The remaining 8.4% appears to be unique to *B. dentium*. The ATG start codon is preferred (78.9% of the time), while GTG and TTG are less frequently used start codons at 18.9% and 2%, respectively.

The presumed origin of replication (*ori*C) of the *B. dentium* Bd1 chromosome, including the adjacent and conserved *dna*A, *dna*N and *rec*A gene configuration, was identified on the basis of common features to corresponding regions in other bacterial chromosomes [30]–[31]. The *ori*C was located proximal to the *dna*A gene, in an AT-rich sequence containing characteristic DnaA boxes, while the position of the replication terminus (*ter*C) was inferred using GC skew analysis (Figure 1).

**Figure 2:**
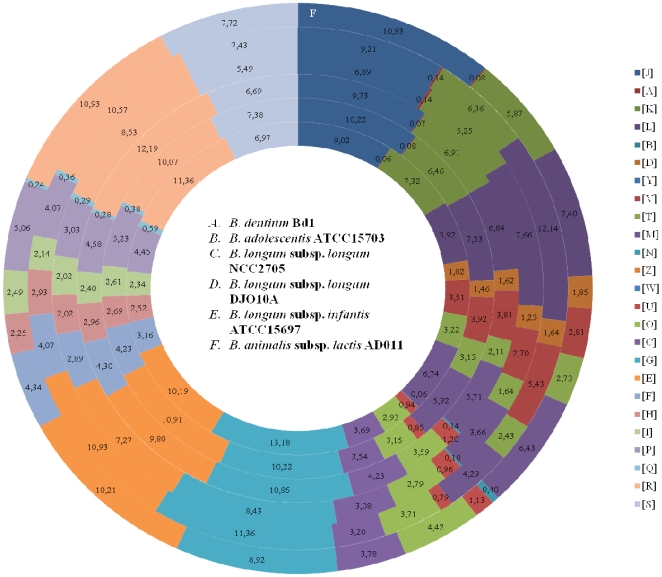
Comparison of COG functional categories between completely sequenced bifidobacterial genomes. Each coloured segment indicates the relative contribution of a functional category as a percentage of total COGs. Each ring indicates a different bifidobacterial genome as labelled. The color of each COG family is indicated in the Figure The name of the bacterial genomes are indicated in the Figure.

The predicted *B. dentium* Bd1 proteins were functionally categorized and the proportions in each category were compared with those of other bifidobacterial genomes (Figure 2). It is notable that approximately 14% of the genes identified in the *B. dentium* Bd1 genome encode proteins that are predicted to be involved in carbohydrate metabolism and transport. Such an extensive genetic adaptation to carbohydrate metabolism is shared, to a similar degree, with enteric bifidobacteria, and likely represents a specific genetic adaptation of bacteria residing in the GIT, apparently both in the upper region (the oral cavity) as well as in the distal tract (the colon) of the GIT.

**Figure 3:**
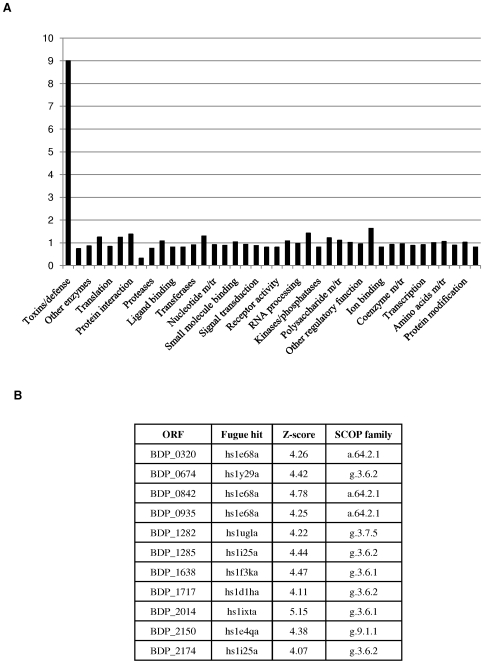
Functional annotation and assignment of the encoded proteins from the genomes of *B. dentium* Bd1 and *B. longum* subsp. *longum* NCC2705 in different superfamily categories according to the Fugue fold recognition method. In (A), each bar represents the odd ratio between *B. dentium* Bd1 and *B. longum* subsp. *longum* NCC2705. The bar corresponding to the toxins/defence superfamily is indicated. In the y-axis is indicated the value of the odd ratio between *B. dentium* Bd1 and *B. longum* subsp. *longum* NCC2705. (B) indicates the different *B. dentium* Bd1 ORFs classified in the toxins/defence superfamily.

Furthermore, 3D-structure prediction of 1955 of the 2143 deduced proteins that constitute the predicted proteome of *B. dentium* Bd1 using the Fugue fold recognition method allowed a functional attribution of these predicted structures by means of the SCOP domain annotation into superfamilies (Figure 3). Such an analysis was also performed for the intestinal *B. longum* subsp. *longum* NCC2705 strain. Notably, both genomes possess a similar protein superfamily content distribution except for proteins assigned to the Toxin-defence group: the *B. dentium* Bd1 genome encodes nine times more proteins with this annotated function as compared to the *B. longum* subsp. *longum* NCC2705 genome (Figure 3). There are 18 genes encoding predicted sensor histidine protein kinases (HPK), 15 of which are located adjacent to a putative response regulator-encoding gene (one of which is separated by just a single gene), distributed throughout the Bd1 chromosome, which is somewhat more than one would predict based on its genome size and the number present in other, similarly sized bifidobacterial genomes (ranging from 5 to 17) [32]. This suggests that the relative abundance of two-component systems (2CSs) in a micro-organism is an indicator of its ability to sense dynamic environmental cues and to modulate appropriate physiological responses, a notion also exemplified by the high number of 2CSs found in *Bacteroides thetaiotaomicron* [33].

### Genome differences in bifidobacteria revealed by genome sequence alignments

**Figure 4:**
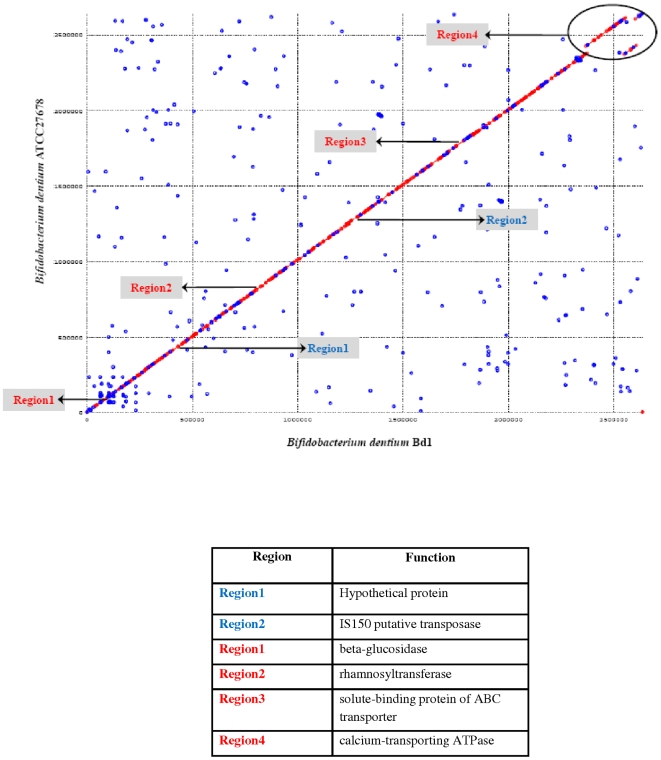
Dot plot of *B. dentium* Bd1 versus *B. dentium* ATCC27678. The visible areas of divergence are indicated. Blue type indicates sequences unique to Bd1 compared with ATCC27678, whereas red type shown sequences absent in Bd1. The deduced functions of the divergent regions are described in the table.

Comparative genomics of intestinal bifidobacteria may elucidate genomic regions involved in the maintenance of physiological homeostasis that is attributed to these bacteria. The genomic structure of *B. dentium* Bd1 is highly syntenic with that of the recently sequenced genome of *B. dentium* ATCC27678 (accession no. ABIX00000000; Figure 4) with an average nucleotide identity of 99% across these two genomes. This Bd1 versus ATCC27678 genome comparison was scrutinized for the identified ORFs with particular consideration of nucleotide changes occurring at particular positions for every codon in the Bd1 genome. The highest substitution rate occurred at the third codon position (36.3% vs. 29% at the second nucleotide and 34.7% at the first nucleotide). Furthermore, a survey of DNA sequence similarity at intergenic regions between both strains revealed a lower level of DNA conservation (98%) compared to that identified between coding regions (99%). Thus, in the *B. dentium* taxa, as is generally the case for *Eubacteria*, the intergenic regions have experienced a higher rate of nucleotide substitution compared to that of the coding regions.

Furthermore, sequence identity varied between the ORFs shared by both genomes (2133); with the large majority displaying an identity of 100%, and just 19 ORFs showing a similarity of less than 95%. The two genomes were shown to contain an identical repertoire of prophage-like and IS elements, although a small number of nucleotide differences (e.g., deletions or substitutions) were noticed for two IS elements, isblo10 and isblo3-2, and for the prophage-like elements Bdent-1 and Bdent-2, suggesting that these strains are very closely related but genetically distinct.

Points of disruption of the gene conservation between the two *B. dentium* genome sequences corresponds to the presence or absence of integrated elements (IS elements) and genes with a predicted function in sugar metabolism (Figure 4). For example, the *B. dentium* ATCC27678 genome contains a putative rhamnosyltransferase-encoding gene, which is located close to an IS element, and which is lacking in the corresponding position on the *B. dentium* Bd1 genome. This suggests that this genetic element in *B. dentium* ATCC27678 was acquired by HGT or it may be lost due to the presence of this mobile element. The conserved gene order was not limited to *B. dentium* ATCC27678, but can be expanded to *B. adolescentis* ATCC15703 (Figure S2). The degree of alignment between the genomes of different bifidobacterial species varied depending on the phylogenetic distance of the genomes being compared. Thus, the alignments of *B. dentium* Bd1 with the genomes of *B. longum* subsp. *longum* NCC2705, *B. longum* subsp. *longum* DJO10A or *B. longum* subsp. *infantis* ATCC15697 display an clearly reduced colinearity, resulting in an X-shaped plot diagram (Figure S2). This is indicative of multiple large rearrangements around the origin-terminus axis of the genome following divergence from a common ancestor [34]. When the genome of a *Bifidobacterium* strain outside the *B. longum* and *B. adolescentis* phylogenetic groups (e.g., *B. animalis* subsp. *lactis* ADO011) was thus aligned, sequence identities were restricted to very small genome segments (Figure S2). The fact that long-range genome alignments of *Bifidobacterium* could not be produced at the DNA level is a significant indication of profound intra-genus diversity, similar to that found for the lactobacilli [35], but contrasting for example with DNA-DNA interspecies alignments among other genera belonging to the *Actinobacteria* [1].

Genome alignments using PROmer allows the reconstruction of broad phylogenetic relationships between prokaryotic genomes [35]–[37]. A previous phylogenomics analysis based on 123 protein sequences representing the minimal core proteins of the *Actinobacteria* phylum highlighted the relatedness of *B. longum* subsp. *longum* NCC2705 to propionibacteria, *Leifsonia* and *Tropheryma* [1]. Here, we included in such an analysis the *B. dentium* Bd1 and other bifidobacterial genome sequences published to date. Interestingly, the resulting neighbour-joining tree revealed a clear evolutionary split of these bifidobacterial sequences with those derived from *Propionibacterium acnes*, *Leifsonia xyli* subsp. *xyli* and *Tropheryma whipplei* (Figure S3), and indicating that bifidobacteria are derived from a deep ancestor of the *Actinobacteria* phylum.

A comparative study was undertaken to determine putative orthology between the *B. dentium* Bd1 CDSs with those of five other completely sequenced bifidobacterial genomes, resulting in 908 putative orthologs that were shared between all these genomes (Figure S4). The most common functional classes represented by these core proteins were those involved in housekeeping functions including information processing, DNA replication, repair, cell division, transcription, translation and secondary metabolite biosynthesis, transport and catabolism. Proteins belonging to functional categories representing sugar and amino acid metabolism, and uptake by ABC transporters were the second largest commonly found group, emphasizing their apparent importance to bifidobacteria (Figure 3). When the genome sequence of *B. dentium* ATCC27678 was included in this analysis a total of 692 CDSs were found that have no matches in currently available bifidobacterial genomes, thus representing *B. dentium*-specific proteins. Over half of these are hypothetical proteins, whereas the remainder have their best matches in sequenced members of the *Actinobacteria* and/or *Firmicutes*, including bacteria of the oral microbiota such as *Actinomyces* spp., *S. mutans* and *Treponema denticola* [38]–[39]. Notably present among these *B. dentium*-specific genes are two adjacent ORFs (BDP_1871- BDP_1872) with homology to the *hip* operon found in *Enterobacteria*, which allows increased survival following various stress conditions [40]. Thus, the *B. dentium hip* operon may positively influence persistence in the oral environment upon exposure to stress conditions, e.g. the fluctuating acid environment that accompanies caries initiation. A subset, i.e. 181, of the 692 *B. dentium*-specific proteins are conserved in both *B. dentium* genomes but do not have significant matches in other currently available genome sequences, and may thus be responsible for certain unique adaptive properties. When these hypothetical proteins were scanned against a database of structural profiles using the FUGUE program, which can recognize distant homologues by sequence-structure comparisons [41], we identified a number of potential homologs with a significant Z-score subdivided in various clusters according to their predicted gene function (Figure S5). Interestingly, this analysis revealed that part (13.33%) of these *B. dentium*-specific proteins clustered in the toxin/defence family, suggesting that these proteins provide protection against host defensins, such as cationic and cysteine-rich peptides [42].

### The mobilome of *B. dentium* genome

**Figure 5:**
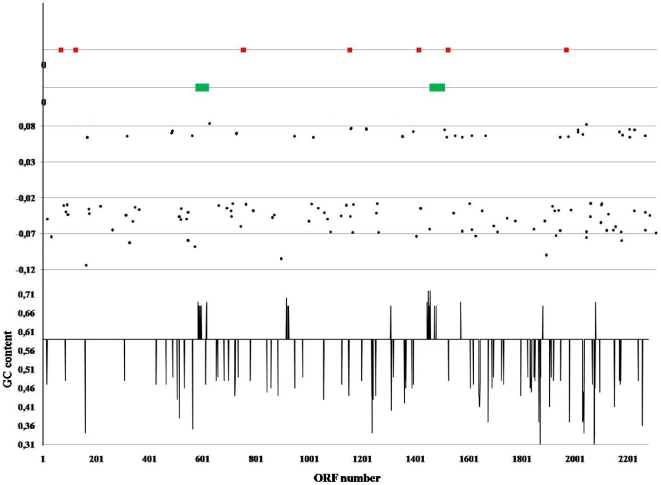
Mobile genetic elements of the *B. dentium* Bd1 genome. IS elements and predicted prophage-like elements are labelled in red and green, respectively. The first plot from the bottom indicates the deviation of the G+C content of each ORF of the *B. dentium* Bd1 genome from the mean average (58.54%). In the second plot each dot represents an ORF displaying a biased codon usage determined by factorial correspondence analysis of codon usage.

The coexistence of *B. dentium* within the oral biofilm which consists of over 900 taxa may facilitate exchange of genetic information that is mediated by mobile genetic elements. Such elements are present in virtually all bacterial genomes, and in some organisms the associated genes may contribute to the metabolism or pathogenic potential of the organisms. Analysis of the *B. dentium* Bd1 genome revealed the presence of conventional mobilome candidates that may have been acquired through Horizontal Gene Transfer (HGT). Analysis of G+C content (G+C), amino acid usage [43], BLASTP best-match and codon preference of the *B. dentium* Bd1 chromosome indicated that considering its total DNA content just 93,300 bp of the *B. dentium* Bd1 genome display a significant deviation (>2-fold difference) from the average values of the paramethers as indicated above and may have been recently acquired by HGT (Figure 5). This suggests that in contrast to other bifidobacterial genomes [44], HGT is not the main force driving genome evolution in *B. dentium* species.

Some representative mobile elements as well as DNA regions acquired by HGT will be discussed below. In *silico* analysis of the *B. dentium* Bd1 genome revealed the presence of two prophage-like elements designed Bdent-1 and Bdent-2, which exhibit a close phylogenetic relationship with phages infecting bacteria belonging to the *Firmicutes* phylum (Ventura et al, AEM in press, published now). The *B. dentium* Bd1 chromosome harbours seven insertion sequences (IS), belonging to three IS families, ISL3, ISL10, ISSdel and IS3 like (Table 1), a number which is much lower than those in other sequenced bifidobacterial genomes (data not shown).

**Table 1:** Genome features of *B. dentium* Bd1.

Trait	Number/value
Size (Mb)	2,636,368
G+C content	58.54%
Number of identified ORF	2143
Assigned function	1684
- Amino acid transport and metabolism^a^	259
- Carbohydrate transport and metabolism^a^	286
- Transcription^a^	174
- Translation^a^	159
- Replication, recombination and repair^a^	123
- Defence mechanisms^a^	60
- Signal transduction^a^	100
- Cell wall/membrane biogenesis^a^	124
- Post-traslational modification, protein turnover, chaperones^a^	55
- Energy production and conversion^a^	70
- Nucleotide transport and metabolism^a^	64
- Coenzyme transport and metabolism^a^	55
- Lipid transport and metabolism^a^	51
- Inorganic ion transport and metabolism^a^	119
Phage regions	2
IS transposase families	3
- ISL3	4
- IS3-like	1
- ISL10	1
- ISSdel	1
CRISPR	2
Fimbrial systems	10
Transporters	771
- ABC systems	298
- PTS systems	2

a According to the COG families.

Additional putative mobile elements identified in the *B. dentium* Bd1 genome are represented by two Clustered of Regularly Interspersed Short Palindromic Repeats (CRISPR) loci, named CRISPR1 and CRISPR2, with adjacent CRISPR-associated *cas* genes, CRISPR-Cas1 and CRISPR-Cas2, respectively. When CRISPR-Cas1 and CRISPR-Cas2 were compared to identified lactic acid bacteria CRISPR loci [45], they clustered into two different CRISPR families, Blon1 and Llel1, respectively (Figure S6), suggestive of two independent HGT events. CRISPRs represent the most widely distributed prokaryotic family of repeats [46],[47], and act as defence systems against invasion of foreign genetic material, in particular phages [48],[49].

### Genome diversity in *B. dentium*

**Figure 6:**
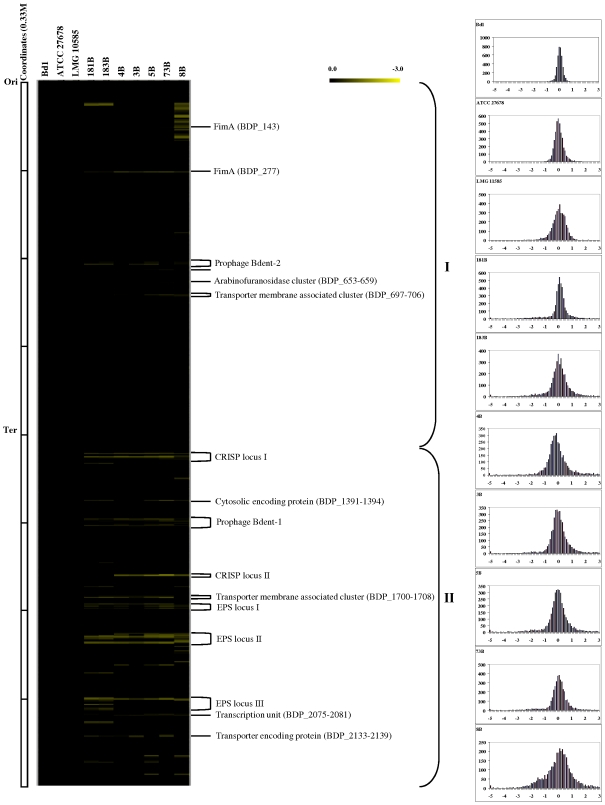
Genomic diversity in the *B. dentium* species with reference to the *B. dentium* strain Bd1 genome (Left) CGH data. Each horizontal row corresponds to a probe on the array, and genes are ordered vertically according to their position on the Bd1 genome. The columns represent the analysed strains, and strains are identified by their code numbers. The colour code corresponding to the presence/absence is given at the top right of the figure: the gradient goes from black to green to indicate the presence, divergence or absence of a gene sequence. The predicted function of some relevant genes are shown on the right-hand margin, ori: origin of replication; ter, terminus of replication (left-hand inset). Right-hand inset displays Signal ratio distribution of the CGH data. The reference is *B. dentium* strain Bd1. Ratios are expressed in a log_2_ scale. See text for details.

The genome variability among different strains of *B. dentium* was investigated by Comparative Genomic Hybridization (CGH) experiments using *B. dentium* Bd1-based microarrays. We determined which and how many ORFs from the sequenced *B. dentium* Bd1 strain did or did not hybridize with total genomic DNA extracted from ten *B. dentium* strains from different origins (dental caries from adult or child, from saliva and from fecal samples). Overall, DNA from the tested *B. dentium* strains failed to efficiently hybridize to between 1% and 12% of the probes from the reference *B. dentium* strain Bd1. These values are small compared to those described for other bacterial species [50]–[56], including bifidobacteria, such as *B. longum* subsp. *longum* [57]. Such findings suggest that the *B. dentium* genome is only slowly evolving compared to other bacteria, including bifidobacterial species residing in the distal tract of the human GIT. Nevertheless, CGH cannot identify regions present in the tested strains but absent from the *B. dentium* Bd1 strain, while it also does not analyze the synteny of the genome. Consequently, caution needs to be employed when applying the term “divergent” to CGH studies. When projected on the genome map of *B. dentium* Bd1, the CGH results highlight clustering of conserved and variable ORFs (Figure 6). The region between the origin of replication and the terminus of replication in the clockwise direction represents the largest genome segment of relative high gene conservation (denoted as I ). In contrast, the region between the replication terminus and the origin of replication in the clockwise direction was shown to be a major area of genetic diversity (II in Figure 6). According to the Bd1 gene annotation, the types of genomic diversity thus identified can be assigned to two classes (i) mobile DNA that constitutes the *B. dentium* mobilome previously identified by *in silico* analyses; (ii) plasticity regions of *B. dentium* genome, which may underlie specific adaptations of the investigated strains, and which could represent laterally acquired DNA or remnants of ancestral DNA that have not (yet) been lost. Plasticity regions which are preferred sites for acquisition of strain-specific DNA are well recognized in the genomes of pathogens like *H. pylori* [58], where array-based CGH has similarly been used to highlight regions involved in adaptation to different pathological roles [59]. Five large DNA segments, which are conserved in *B. dentium* Bd1 and in the closely related strains ATCC27678 and LMG10585, clearly represent mobile DNA: two prophage-like elements, Bdent-1 and Bdent-1, the CRISPR elements and the cytosolic proteins (BDP_1391–1394) (Figure 6). In some strains, stretches of hybridizing prophage genes matched individual modules of the prophage (Figure 6, Bdent-2 prophage-like element), an observation which agrees with the hypothesis of modular phage evolution [60]. Within the variable regions of the CGH map, indicated as plasticity regions, genes associated with bacterium-environment interaction and metabolic abilities appear to be particularly enriched. These include the *eps* clusters, a putative fimbrial-biosynthesis gene cluster and membrane-associated transporters. The *eps* clusters of the Bd1 strain are associated with the dTDP-rhamnose biosynthesis locus, and represent the largest genome segment with substantial inter-strain genetic variability.

When the CGH data are expressed on a log_2_ scale according to the mean ratios of the normalized results, a major peak is noticed at the same position for all the *B. dentium* strains tested, indicative of very similar DNA sequences (Figure 6A–6L). In addition to the inset of Figure 6, which globally quantifies the similarity of the test strains versus *B. dentium* Bd1, a clustering of the microarray data was performed in order to extract qualitative information about the presence of each gene. A phylogenetic tree based upon these CGH scores identified *B. dentium* ATCC27678 and *B. dentium* LMG 10585 as the closest relatives of *B. dentium* Bd1 (Figure S7). Moreover, CGH clustering produced four groups of *B. dentium* strains based on varying levels of genetic diversity, and largely corresponding to their ecological origin (Figure S7). As a complement to the CGH analysis, we performed multilocus sequence analyses for the same strains, using the genes for the Clp ATPase (*clp*C gene), two F6P-phosphoketolases (*xfp* gene), DnaJ chaperone (*dna*J1), and DNA-directed RNA polymerase B' subunit (*rpo*C gene) as phylogenetic markers [61]. As expected, the phylogenetic tree produced from these concatenated sequences confirmed the clustering of GCH based data (Figure S7).

### Metabolism and transport of *B. dentium*

Homologs of all the enzymes necessary for the fermentation of glucose and fructose to lactic acid and acetate through the characteristic “fructose-6-phosphate shunt” [62], as well as a partial Embden-Meyerhoff pathway were annotated in the *B. dentium* Bd1 genome. These metabolic pathways are important for generation of pyruvate and re-oxidation of NADH, as well as for synthesis of an additional ATP molecule per glucose during the conversion of pyruvate to acetate. The enzymes responsible for pyruvate metabolism identified in the *B. dentium* genome include xylulose 5-phosphate/fructose-6-phosphate phosphoketolase, pyruvate dehydrogenase, pyruvate formate-lyase, phosphotransacetylase, acetate kinase, lactate dehydrogenase. *B. dentium* Bd1 possesses an incomplete tricarboxylic acid (TCA) cycle, which lacks oxoglutarate dehydrogenase, fumarase and malate dehydrogenase. The primary role of these TCA enzymes is most likely the production of precursors for amino acid and nucleotide biosynthesis. Since *B. dentium* Bd1 can be cultured anaerobically with urea, arginine and cysteine as the sole nitrogen sources (unpublished data), it was not surprising that genes required for the biosynthetic pathways of all amino acids were identified in the genome. The way in which cysteine is synthesized is unclear, as the genes involved in sulphate/sulphite assimilation are not present in the *B. dentium* Bd1 genome. It may synthesize cysteine in a manner similar to that suggested for *B. longum* subsp. *longum* NCC2705 using homologs of the genes for cysteine synthase/cysthathione beta synthase, O-acetylhomoserine aminocarboxypropyltransferase and cystathionine γ-synthase, and utilizing a reduced sulphur-containing compound as a sulphur source [23]. Genes encoding complete biosynthetic pathways for purines and pyrimidines from glutamine, as well as for riboflavin, thiamine and folate were identified, while no homologues were present for pathways to produce biotin, pyridoxine, cobalamin, panthotenate and niacin/nicotinic acid, which are also variably distributed in the genomes of other sequenced bifidobacterial genomes (data not shown).

Comparative analysis against the Transport and Classification Database [63] predicts that the *B. dentium* Bd1 genome contains 771 genes encoding (components of) transport systems, accounting for almost 34% of the total number of ORFs (Figure S8A).

Transport in *B. dentium* Bd1 is largely carried out by transporters or carriers (e.g., uniporters, symporters and antiporters) and by P-P-bond hydrolysis-driven transporters. A large proportion of the identified transporters are ATP-dependent, as expected for a microorganism lacking an electron transport chain [64]. Annotated solute-transporting ATPases include P-type, F-type and ABC-type. The P-type ATPases are predicted to be involved in the transport of calcium and potassium, whereas the F-type ATPases (e,g., F_0_F_1_ATPases) use an electrochemical gradient of H^+^ or Na^+^ to synthesize ATP, or hydrolyze ATP to reverse the electrochemical gradient [65]. As described below, a single predicted H^+^-transporting ATP synthase-ATPase is encoded by the *B. dentium* Bd1 genome. We identified 298 predicted ABC-type ATPases, of which about 70% are categorized as importers, representing the most abundant transport category, and accounting for almost 13% of all *B. dentium* Bd1 gene products. The ABC transporters identified have a predicted specificity for a wide variety of substrates, including amino acids, carbohydrates, oligopeptides, osmoprotectants (e.g., proline/glycine, betaine, choline), inorganic ions (e.g., Fe3+, Co2+, Mn2+, phosphate, nitrate, sulphate, and molybdenum) and antimicrobial peptides.

The vast majority of carbohydrate-modifying enzymes encoded by *B. dentium* Bd1 are predicted to be intracellular and so the uptake of sugars with a low degree of polymerization is a key component of *B. dentium* carbohydrate metabolism. The genome of *B. dentium* Bd1 encodes at least 167 ABC transport systems for dietary carbohydrates (Table S1). The *B. dentium* Bd1 genome also specifies two phospoenolpyruvate-phosphotransferase systems (PEP-PTS), consisting of the two general energy-coupling components, enzyme I (EI) and a heat-stable protein (Hpr), and two different sugar-specific multiprotein permeases known as enzyme II (EII).

### Adaptation to the oral cavity

**Figure 7:**
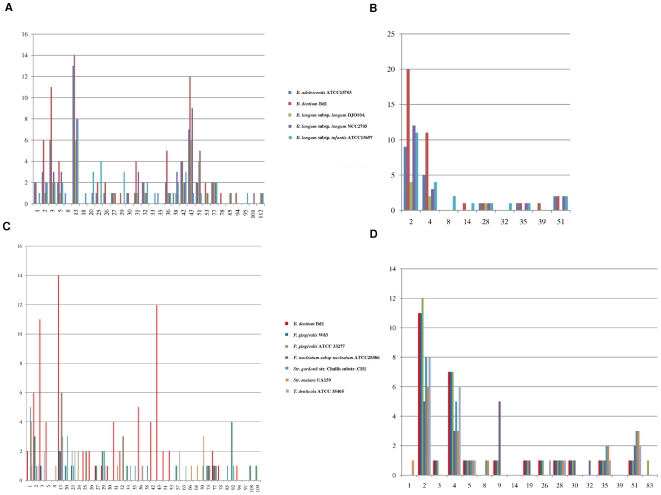
Analysis of the glycobiome of the *B. dentium* Bd1 genome by reference to CAZy database. (A) The glycoside-hydrolase (GH) families identified in the genome of *B. dentium* Bd1 and in enteric bifidobacterial genomes. (B) The glycosyl-transferase families (GT) encoded by the genome of *B. dentium* Bd1 and by enteric bifidobacterial genomes. (C) The GH families identified in the genome of *B. dentium* Bd1 and in other oral pathogens. (D) The GT families identified in the genome of *B. dentium* Bd1 and in other oral pathogens. In each panel, the X-axis represents the different GH families or GT families according to the CAZy database (Henrissat 1999), whereas the Y-axis indicates the abundance of each GH family expressed in percentage.

The human oral cavity is a complex microbial ecosystem, the composition of which may vary depending on the frequency and nature of food ingestion with consequent fluctuations in biofilm pH. Compared to the distal bowel, where organisms are presented with a relatively consistent stream of molecules that cannot be metabolized or degraded by the more proximal microbiota, the oral cavity microbiota is exposed to the full contents of the ingested foods. Thus, possessing extensive catabolic abilities for carbohydrates is a potent energy-harvesting mechanism for *B. dentium* Bd1. Genomic data combined with our own data suggests that *B. dentium* Bd1 has a significantly larger arsenal of genes allowing for breakdown of sugars, also called glycobiome [44], as compared to other bifidobacterial species [23]–[25] or other characterized members of the oral microbiota (Figure 7C and 7D). Classification according to the Carbohydrate Active Enzymes (CAZy) system of Coutinho & Henrissat (1999) showed that the Bd1 genome specifies 117 carbohydrate-active genes including glycoside-hydrolases (GH), glycosyl-transferases (GT) and glycosyl-esterases (CE), which are distributed in 27 GH families, seven GT and three CE families (Figure 7A and 7B). The majority of the identified GH enzymes from *B. dentium* Bd1 are predicted to be intracellular, with a putative cellulase (BDP_2148) and a xylosidase (BDP_0236) predicted to be the only extracellular GH enzymes. Members of GH families that had previously not been detected in bifidobacterial genomes are GH78 (BDP_2152) and GH94 (BDP_2127), which are predicted to be involved in the metabolism of fucose (GH78) and cellobiose (GH94). Furthermore, the Bd1 genome encodes a wide variety of enzymes to ferment different pentose sugars (e.g., xylose, ribose and arabinose).

The fermentation abilities of this oral strain are clearly broader than those of the phylogenetically related enteric *B. adolescentis* ATCC15703 (Figure S9), probably reflecting the ecological niche it occupies, which apparently contains a higher variety of available sugars as compared to the distal regions of the gastrointestinal tract.

In addition to the transient food components, the human oral cavity is coated with large amounts of viscous secretion produced by the acinar cells of the salivary glands. This secretion consists predominantly of a heterogeneous population of glycoproteins, commonly referred to as salivary mucins [66]. These large, heavily glycosylated glycoproteins play a major role in the maintenance of viscoelastic properties of saliva, participate in the formation of the protective oral mucosal mucus coat and tooth enamel pellicle [67]. Salivary mucins are comprised of 20–22% protein, 0.2% covalently bound fatty acids, and 68–72% carbohydrate [66]. Notably, the carbohydrate component consists mainly of fucose, mannose, galactose (Gal), N-acetylglucosamine (GlcNAc) and N-acetylgalactosamine (GalNAc). The *B. dentium* Bd1 genome contains an extensive gene repertoire that appears to be dedicated to the metabolism of the backbone of mucin-containing carbohydrate structures, such as Galβ-1,3-GalNAc or Galβ-1,4-GlcNAc disaccharides. This repertoire includes genes for predicted enzymes such as a glucosaminidase and β-galactosidase that could be involved in the removal of monomeric carbohydrates from mucins. Moreover, the presence of a gene encoding a putative fucosidase enzyme (BDP_2152) indicates that *B. dentium* Bd1 can probably degrade fucose-containing glycans, such as those present in salivary mucins [68].

Salivary glycoproteins also contain considerably quantities (3.8–4%) of sialic acid and sulphate which decorate the surface of the mucin sugar backbone [66]. Interestingly, a predicted O-sialoglycoprotein endopeptidase (BDP_1212) and a sialic-acid specific acetylesterase (BDP_0122) were annotated in the genome of *B. dentium* Bd1. However, genes encoding a sialidase and additional enzymes to degrade sialic acid, which have been identified in other bifidobacterial genomes such as *B. longum* subsp. *infantis* ATCC15697 [25], do not appear to be present in the genome of *B. dentium* Bd1.

Dental caries is initiated by demineralization of the tooth surface due to the action of organic acid formed by dental plaque bacteria, arising from their fermentation of dietary carbohydrates. After fermentable carbohydrate intake, the plaque pH may decrease below the critical pH of 5.5, at which point human enamel undergoes demineralization, within minutes, and may remain acidified for several minutes up to several hours [69],[70]. This rapid acidification may not only cause demineralization of tooth surface but also temporarily inhibit bacterial growth in the oral biofilm. Thus, a high level of inherent acid tolerance appears to be crucial for the cariogenicity of oral microorganisms [71]. When the intracellular pH maintained by *B. dentium* under varying external pH conditions was experimentally compared to those of other caries-associated oral bacteria, such as *Str. mutans* and *Lactobacillus paracasei*, *B. dentium* Bd1 displayed a superior ability to keep a more neutral internal pH compared to these two other bacteria (Figure S10A and Nakajo, Takahashi and Beighton, personal communication). Moreover, when *B. dentium* Bd1 was cultivated in a synthetic medium at different pH values the growth of Bd1 was not significantly reduced by the highest level of acidity tested (pH 4), a value which can be reached in the oral cavity after food ingestion (Figure S10B). Notably, other closely related bifidobacteria which occupy a different ecological niche (intestinal vs. oral) do not exhibit this aciduric property (Figure S10B and data not shown). Higher levels of inherent tolerance of oral bacteria to acidification have been related to the presence of a membrane-bound, acid-stable, proton-translocating F_1_F_0_ ATPase system whose activity has been considered crucial in maintaining the intracellular pH at 7.5 [71]. In the *B. dentium* Bd1 genome, the F_1_F_0_-ATPase is encoded by the *atp* operon, but this system is also encoded by other bifidobacteria [72]. However, the genome of *B. dentium* Bd1 contains two adjacently located genes (BDP_1749 and BDP_1750) encoding a glutamate decarboxylase (GadB) and a glutamate/gamma-aminobutyrate anti-porter (GadC), not present in other bifidobacterial genomes so far published [23]–[25], and known in other bacteria to form a glutamate-dependent acid resistance system 2 (AR2) [73].

### Transcriptional profiling analysis of *B. dentium* Bd1 and adaptation to the oral lifestyle

Characteristics contributing to the ecological fitness in the oral cavity, such as utilization of different diet-derived carbohydrates, and stress tolerance to antimicrobial compounds and acidic environments, should be discernible in *B. dentium* Bd1. To determine if *B. dentium* Bd1 functionally responds to stressful stimuli, we performed transcriptional profiling studies using Agilent arrays (Agilent, Palo Alto, Ca., USA) that contain oligonucleotides representing 2114 of the 2143 predicted *B. dentium* Bd1 protein-encoding genes.

#### (i) Identification of genes differentially expressed upon exposure to acidic environments

**Figure 8:**
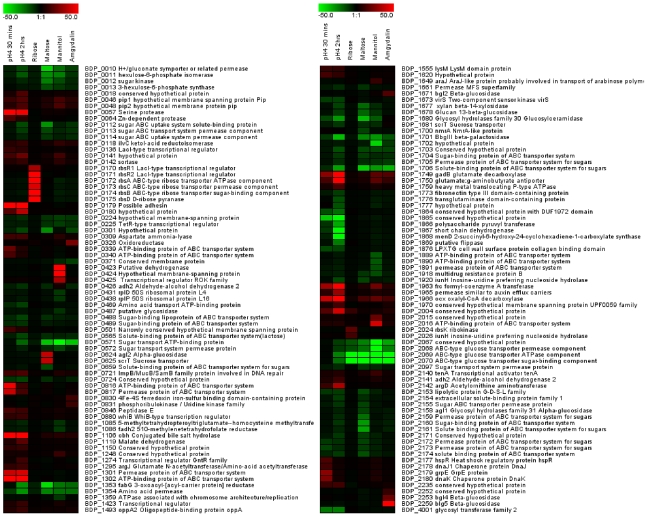
Identification of *B. dentium* Bd1 transcribed genes by DNA–micro array analysis. The heat-map indicates the change in the expression upon cultivation of Bd1 cells at low pH as well as represents selected genes that were up- or down-regulated when grown in media containing various carbohydrates as the sole carbon sources as compared to growth on glucose. Each row represents a separate transcript and each column represents a separate sample. Colour legend is on the top of each micro array plot, red indicates increased transcription levels, whereas green indicates decreased trasncription level as compared to the reference samples (glucose-grown samples or growth at pH 6).

To study changes in gene expression occurring in *B. dentium* Bd1 in response to acidic conditions, cultures were exposed to acidic conditions simulating those occurring in active carious lesions (pH 4). In a time course experiment the global gene expression of *B. dentium* Bd1 cells exposed to a pH change from 7.0 to 4.0 was compared to gene expression of untreated cells. Samples were collected 30 minutes and 2 hours after the onset of acid stress, and submitted to transcriptome analysis. The complete list of the genes whose expression pattern was altered following acidic exposure is presented in Figure 8 and Table S2. In total, 46 genes displayed a profound change in mRNA expression levels (greater than tenfold) in response to acid stress. Genes BDP_1749 and BDP_1750, encoding a glutamate carboxylate and a glutamate: γ-aminobutyrate antiporter were upregulated 90 and 51 fold, respectively, consistent with their role in acid stress (see above). Also upregulated were genes encoding aspartate ammonium lyase (BDP_0309), formyl-coenzyme A transferase (BDP_1963), oxalyl-CoA decarboxylase (BDP_1966) and malate dehydrogenase (BDP_1119), all known to provide mechanisms to cope with acid stress [74]. Additionally, genes involved in arginine (i.e. ArgD) or glutamate metabolism were upregulated, perhaps linking this to the deamination of branched chain amino acids (BCAA) as a mechanism of maintaining the internal pH of the cells [75]. Furthermore, acetylornithine aminotransferase (ArgD) catalyzes a reaction that could serve as a way of recycling the 2-oxoglutarate formed by the BCAA aminotransferase as well as feeding glutamine synthesis.

The bile salt hydrolase-encoding gene, BDP_1106, was shown to be upregulated 97 fold after prolonged acid stress. It is tempting to speculate that *B. dentium* increases its bile salt hydrolase activity in response to imminent exposure to high levels of bile salt when it senses a low pH in its environment (e.g. when exposed to gastric juice during the passage from the oral cavity to the intestine). Other upregulated genes, especially after 2 hrs of acid stress, had in common that they are involved in proteolytic degradation or amino acid uptake or amino acid catabolism. Surprisingly the F_1_F_o_-ATPase, encoded by BDP_1952–1959, was not upregulated, in contrast to what has been observed for *B. animalis* subsp. *lactis* DSM10140 [72]. These data suggest that *B. dentium* manages its acid tolerance by amino acid degradation.

#### ii) Induction of genes related to the utilization of carbohydrates

Genome analysis of *B. dentium* Bd1, as supported by growth tests, revealed extensive genetic capabilities of this strain to ferment carbohydrates (see above and Figure S9). To validate the genetic basis for these observations, we investigated the transcriptome of *B. dentium* Bd1 grown on the most efficiently utilized carbohydrates (glucose, ribose, maltose, mannitol and amygdalin). As expected the transcriptional profiling data revealed induction of genes involved in sugar metabolism (Figure 8 and Table S3). Genes specifically induced when Bd1 was grown on ribose included *rbs*R1, *rbs*R2, *rbs*A, *rbs*B, *rbs*C and *rbs*D encoding transcriptional regulators, ABC transporters and ribose pyranase (Table S3), which are organized in a genetic cluster (BDP_170-BDP_175). In addition, the expression levels of another locus spanning BDP_2024 to BDP_2026, encoding a ribokinase, an inosine-uridine nucleoside hydrolase and a transporter, respectively, were shown to be more than 9-fold higher than their levels in the absence of ribose (Table S3). Maltose afforded excellent growth of *B. dentium* Bd1 and based on transcriptomics data, the genes related to the utilization of this sugar included two adjacent genes (BDP_0624-BDP_0625), which were highly up-regulated, and which encode a putative α-glucosidase and a sugar transporter, respectively (Table S3 and Figure S9).

The transcription of three genes (BDP_2252, BDP_2253 and BDP_2259) were shown to be enhanced when the Bd1 was grown on amygdalin, suggesting their role in the utilization of this complex sugar. Three adjacent genes (BDP_0423-BDP-0425), encoding a mannitol dehydrogenase, a mannitol permease and a transcriptional regulator, were shown to be up-regulated between 32- and 200- fold when Bd1 was grown in the presence of mannitol (Table S3).

#### iii) Evaluation of genes involved in biocide resistance

Oral bacteria are exposed to a large variety of antimicrobials/biocides that are ingested with food (e.g. bacteriocins and food preservatives) and/or are used in normal hygiene practices (e.g., mouth-washes). Transcriptional profiling was performed of the *B. dentium* Bd1 strain grown in the presence of diluted mouth-washes or of the commonly used mouth biocides such as chlorhexidine was performed. In total 112 genes were upregulated (fold >2.5) when cells were exposed to the mouth-wash for 2h or 8h (data not shown). These include genes encoding components of predicted major facilitator systems and extracellular solute binding proteins, which may chelate and extrude the cytotoxic compounds of the mouth-washes. As expected, other stress-related genes were induced upon exposure of Bd1 cells to mouthwash, such as those coding molecular chaperones, aminotransferase and glycosyltransferases. In contrast, only 2 genes were upregulated when the cells were exposed to chlorhexidine, while >1000 genes were downregulated. Presumably exposure to chlorhexidine is lethal, thereby resulting in a drastic decrease of all mRNA. The upregulated genes include a putative multidrug transporter *mdr*B (BDP_1665) (data not shown).

### Putative virulence factors

Although *B. dentium* is not an invasive, life-threatening pathogen it plays a role in tooth tissue destruction and infects tooth dentine, and there are a number of ORFs that code for potential colonization or virulence factors, such as adhesins, exoenzymes, protease- and cytokine-modulating molecules, as well as putative hemolysins (see Table 2 for an overview of putative virulence factors of *B. dentium* Bd1, some of which will be discussed below). The latter are similar to hemolysins from oral pathogens, including Hemolysin A from *S. mutans*, a coiled-coil myosin-like protein. Among the putative *B. dentium* Bd1 surface antigen proteins, the BDP_0164 protein displays 51% similarity to the *T. denticola* pathogen-specific surface antigen, and is flanked on one side by an ORF (BDP_0163) involved in iron metabolism (high-affinity iron permease) and on the other side by a gene (BDP_0165) encoding an integral membrane protein with high similarity to a protein encoded by the oral pathogen *Fusobacterium nucleatum*. This suggests that the gene cluster (BDP_0163-BDP_0165) is involved in iron acquisition and adhesion.

**Table 2:** Putative virulence factors identified in the genome of *B. dentium* Bd1.

General function	Gene	Predicted function
Exotoxins	BD_0019, BDP_0878, BDP_1280	Hemolysin
Adhesins	BDP_0517	Myosin-cross reactive antigen
	BDP_523, BDP_643, BDP_1896_	Surface antigen
	BDP_2059	SpaA adesin
	BDP_1864, BDP_1864a, BDP_2047, BDP_2048	Cell wall polysaccharides biosynthesis
	BDP_2045, BDP_2054, BDP_2056, BDP_2059, BDP_2061	Inter-bacterial aggregation protein
	BDP_0279, BDP_2189	Collagen adhesion proteins
	BDP_1773	Fibronectin/fibrinogen-binding protein
	BDP_535, BDP_1224	FimA
Exoenzymes/proteases	BDP_0549	Zn-dependent protease
	BDP_0375	Collagenase

Oral bacteria can adhere to salivary agglutinin, other plaque bacteria, extracellular matrix and epithelial cell-surface receptors [76]. In the most intensely studied oral pathogen, *S. mutans*, two major adhesins mediate this attachment: cell-surface or adhesion proteins, such as SpaA adhesins [77], and sucrose-derived glucans (e.g., *gbp*B). A homolog (BDP_2059; 32% identity) of the gene encoding a major adhesin of viridans streptococci, SpaA, was identified in the genome of *B. dentium* Bd1 (Table 2). SpaA binds to human salivary agglutinin, collagen and cells of certain oral pathogens, such as *Actinomyces naeslundii* [78],[79]. Notably, the BDP_2059 putative adhesin also contains a domain that is similar to a domain of a *Streptococcus gordonii* protein which mediates strong lactose-inhibitable coaggregation [80]. Furthermore, part of an operon that is required for the synthesis of cell wall polysaccharides in *S. mutans* UA159, i.e. *rgp*A, *rgp*B, *rgp*D and *rgp*C [81],[82], exhibits clear homology with *B. dentium* Bd1 ORFs BDP_1864, BDP_1864a, BDP_2047 and BDP_2048, respectively. In *S. mutans* these genes play not only a crucial role in binding to human oral tissues [83],[84] but they also participate in serotype determination [85]. These genes also show a strong divergence in G+C content relative to the remainder of the genome, indicating that this region has been acquired by horizontal gene transfer.

The *B. dentium* Bd1 genome also specifies surface proteins with domains that resemble those (Pfam number 31902) responsible for inter-bacterial aggregation by a choline-binding domain. Such choline-binding motifs are present in the ligands of the most important pneumococcal virulence proteins, encoded by *psp*C and *psp*A, which presumably act as adhesins that bind to host factors such as IgA and factor H [86]. Notably, and in contrast to the available chromosomes of enteric bifidobacteria, the *B. dentium* Bd1 genome harbors five adjacent genes that encode proteins containing such predicted choline-binding domains (BDP_2045, BDP_2054, BDP_2056, BDP_2059 and BDP_2061).

Furthermore, a large number of predicted surface and extracellular proteins that may be involved in host attachment and interaction were identified in a similar fashion as described for other oral pathogens [87],[88] (Table 2).

**Figure 9:**
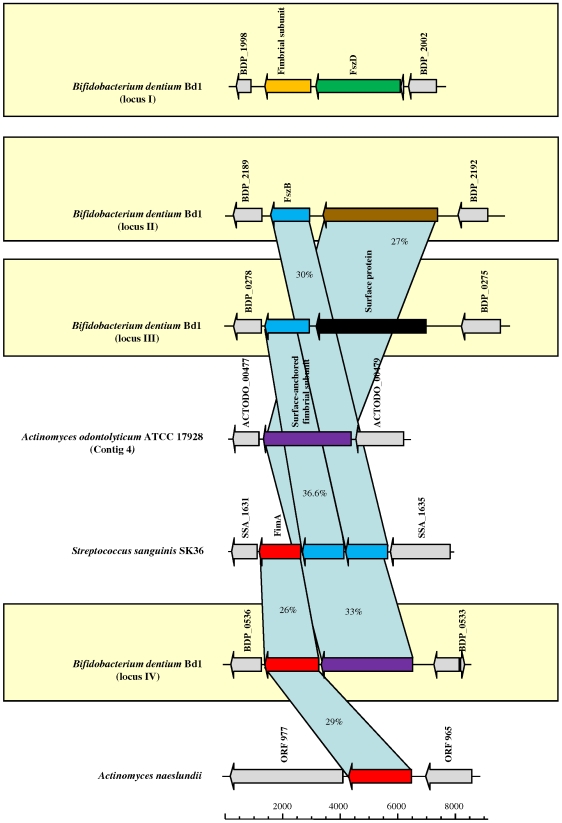
Comparison of presumptive fimbrial loci in *B. dentium* Bd1 with the corresponding loci in different oral bacteria. Each arrow indicates an ORF. The length of the arrow is proportional to the length of the predicted open reading frame. Corresponding genes are marked with the same colour. The putative function of the protein is indicated above each arrow.

Other potential adhesion and virulence factors include glycoprotein-binding fimbriae that, in the oral cavity, may mediate the recognition of and adhesion to salivary proline-rich proteins that bind to tooth and mucosal epithelial cell surfaces. They may also bind to cell wall polysaccharides of certain oral bacteria [89]–[94]. So far bifidobacteria have not been shown to possess any fimbria-like structures on their cell surface, although homologs of fimbrial subunits have been identified in intestinal bifidobacteria [23],[44]. Notably, four loci encoding homologs of known fimbrial subunits FimA, FszB and FszD were identified in the *B. dentium* Bd1 genome (Figure 9). FimA (BDP_0535 and BDP_1224) displays high identity to FimA homologs found in oral commensals such as *A. naeslundii* and *A. odontolyticus* [91],[95].

Finally, *B. dentium* Bd1 encodes a number of putative proteases that may contribute to virulence by their ability to degrade host proteins for bacterial nutrition [96],[97] (Table 2).

## Discussion

We report here the *B. dentium* Bd1 genome sequence that constitutes the first genome based analysis of a bifidobacterial taxon recognized as an opportunistic pathogen. Although a large number of bacteria coexist in the oral cavity and upper respiratory tract in humans they have evolved to form a microbial community with complex physical and biochemical interactions. The analysis of the *B. dentium* Bd1 genome and comparisons with other bifidobacteria residing in the human intestine has revealed insights into the particular evolution and adaptive responses of the opportunistic pathogen *B. dentium* to the oral cavity. *B. dentium*, unlike its intestinal relatives that often are claimed to promote the health-status of their host, contributes to the destruction of the dentition. The elucidation of a putative“cariogenic gene suite” would provide salient targets to test the relative contribution of *B. dentium* to a core cariogenic microbiome, its impact on microbial colonization and succession, and its phylogenetic distribution. It remains unknown if these genomic features constitute a unique competitive strategy evolved in *B. dentium*. As such, it differs from other known bifidobacteria in several aspects of its basic physiology and its adaptation to an ecological niche. As our genome analysis shows, *B. dentium* can metabolize a much larger variety of carbohydrates than other *Bifidobacterium* species sequenced so far and greater than the range of oral streptococcal including *S. mutans*. Genes encoding putative virulence factors associated with adhesins, acid tolerance, defense against toxic substances and capacity in utilizing saliva-derived components, represents genetic evidence of the capacity of *B. dentium* to colonize the oral cavity and to proliferate within active carious lesions. The genome sequence, when explored using functional genomics approaches, will permit the analysis of genes involved in colonization, survival, growth and pathobiology of *B. dentium* in this unique polymicrobial environment.

## Materials and Methods

### 

The strain used in this study *B. dentium* Bd1 is equivalent to the type strain of *B. dentium* species (ATCC27534 or LMG11045 or DSM20436 or JCM 1195).

The genome sequence of *B. dentium* Bd1 was determined by shotgun sequencing and subsequent gap closure (Agencourt Genomic Services, MA, USA). The Bd1 genome was sequenced to approximately 10-fold coverage and assembled with Phred [98], Phrap and the Staden package [99]. Automated gene modelling was achieved using multiple databases and modelling packages as described previously [100]. Additional information on sequencing, bioinformatic, and functional genomics analyses are provided in Text S1.

### Nucleotide sequence accession numbers

The sequence reported in this article has been deposited in the GenBank database (accession number CP001750).

## Supporting Information

Figure S1 Schematic representation of the GC content bias in 696 genomes. The *B. dentium* Bd1 GC content is circularized.
(0.34 MB TIF)

Figure S2 Genome colinearity of *B. dentium* Bd1 with *B. adolescentis* ATCC15703 (A), *B. longum* subsp. *infantis* ATCC15678 (B), *B. longum* subsp. *longum* DJO10A (C), *B. longum* subsp. *longum* NCC2705 (D), and *B. animalis* subsp. *lactis* ADO11 (E). Each dot matrix was calculated using MUMmer. The comparison window was 50 bp and the stringency was 30 bp.
(1.80 MB TIF)

Figure S3 Phylogenetic supertree based on the sequences of *Actinobacteria* core proteins, using SplitsTree.
(0.29 MB TIF)

Figure S4 Venn diagram of homologs shared between sequenced bifidobacterial genomes. Circle sizes are proportional to members contained in each set.
(0.31 MB TIF)

Figure S5 Analysis of the proteins encoded by the genome of *B. dentium* Bd1 according to the distribution of functions in terms of SCOP Domain Superfamilies. (A) shows a pie chart displaying the proportion of proteins encoded by the genome of *B. dentium* Bd1 classified according to general functional categories, while (B) shows a pie chart distribution of the proteins encoded by the genome of *B. dentium* Bd1 when classified according to more detailed functional categories.
(0.47 MB TIF)

Figure S6 Comparative analysis of Cas1 protein sequences. CRISPR repeat families are indicated within the shaded boxes. Bootstrap values are indicated at the nodes for a total of 1,000 replicates. The arrows pointed the Cas protein in the two CRISPR loci of *B. dentium* Bd1. Bootstrap values above 40 are shown.
(0.39 MB TIF)

Figure S7 Polyphasic analysis of the genetic diversity in the *B. dentium* species using the CGH clustering data and a phylogenetic tree of the ten *B. dentium* strains computed from the concatenation of *clpC*, *dnaJ1*, *rpoC*, and *xfp* gene sequences by the neighbour-joining method and Kimura's two parameter model as the substitution model. In each tree, the strain is indicated at the right end of the branch, the colour typing indicates the different ecological origin, i.e., red from dental caries, green from saliva of patients with caries, blue from saliva of healthy patients and yellow from fecal samples. The numbers at the nodes relate to the bootstrap probabilities. The different clusters are boxed.
(0.34 MB TIF)

Figure S8 Predicted transport capabilities of *B. dentium* Bd1 compared to other bifidobacteria (A). Predicted compounds transported by the sequenced bifidobacteria (B).
(0.43 MB TIF)

Figure S9 Carbohydrate metabolizing capabilities of *B. dentium* Bd1. (A) shows the sugar fermentation profiles of *B. dentium* Bd1 and *B. adolescentis* ATCC15703 strains, respectively. Carbohydrates used are indicated. + indicates acid production; − indicates absence of acid production. (B) displays the growth curves of *B. dentium* Bd1 on different carbohydrates as their sole carbon source. The carbohydrates used are indicated next to each curve.
(6.37 MB TIF)

Figure S10 Ecological adaptability of *B. dentium* Bd1 to acidic environments. (A) indicates the intracellular pH of *B. dentium* Bd1, *S. mutans* UA159, and *Lb. paracasei* subsp. *paracasei* ATCC11974 at various extracellular pH values. The data obtained from three independent experiments were plotted. In (B), growth of *B. dentium* Bd1 and *B. longum* subsp. *longum* ATCC 15707 cultures maintained at different acidic conditions were monitored over 24 hours. The colour of the line indicates the pH value of the medium used: red, pH 5; blue, pH4; green, pH 3.
(0.90 MB TIF)

Table S1 Number of ABC exporter and importer system according to substrate type present in the bifidobacterial genome sequenced so date.
(0.05 MB DOC)

Table S2 Selected genes upregulated/downregulated upon acidic stress.
(0.07 MB DOC)

Table S3 Selected genes differentially expressed upon *B. dentium* Bd1 growth in different sugar-based media relative to growth in glucose.
(0.09 MB DOC)

Text S1 Predicted interactions between *B. dentium* Bd1 and host, genetic regions of oral pathogens present in the *B. dentium* Bd1 genome and detailed description of the experimental procedures.
(0.13 MB DOC)
