# Supplementary figures and images for: The Bifidobacterium dentium Bd1 Genome Sequence Reflects Its Genetic Adaptation to the Human Oral Cavity
Source: PLoS Genet. 2009 Dec 24;5(12):e1000785. doi: 10.1371/journal.pgen.1000785 (PMC2788695; doi:10.1371/journal.pgen.1000785)

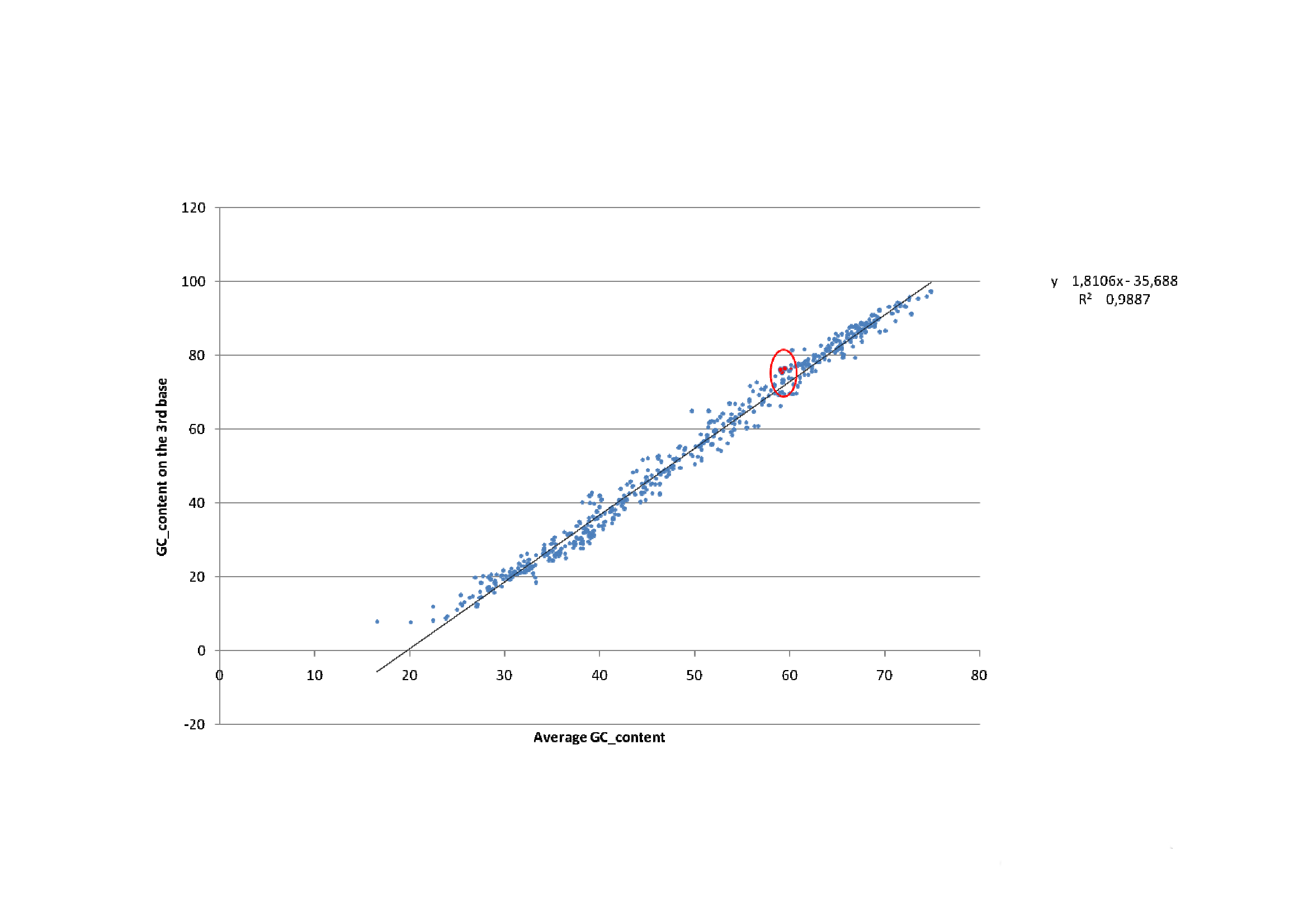

Supplement: Figure S1 — Schematic representation of the GC content bias in 696 genomes. The B. dentium Bd1 GC content is circularized. (0.34 MB TIF) [file pgen.1000785.s001.tif]

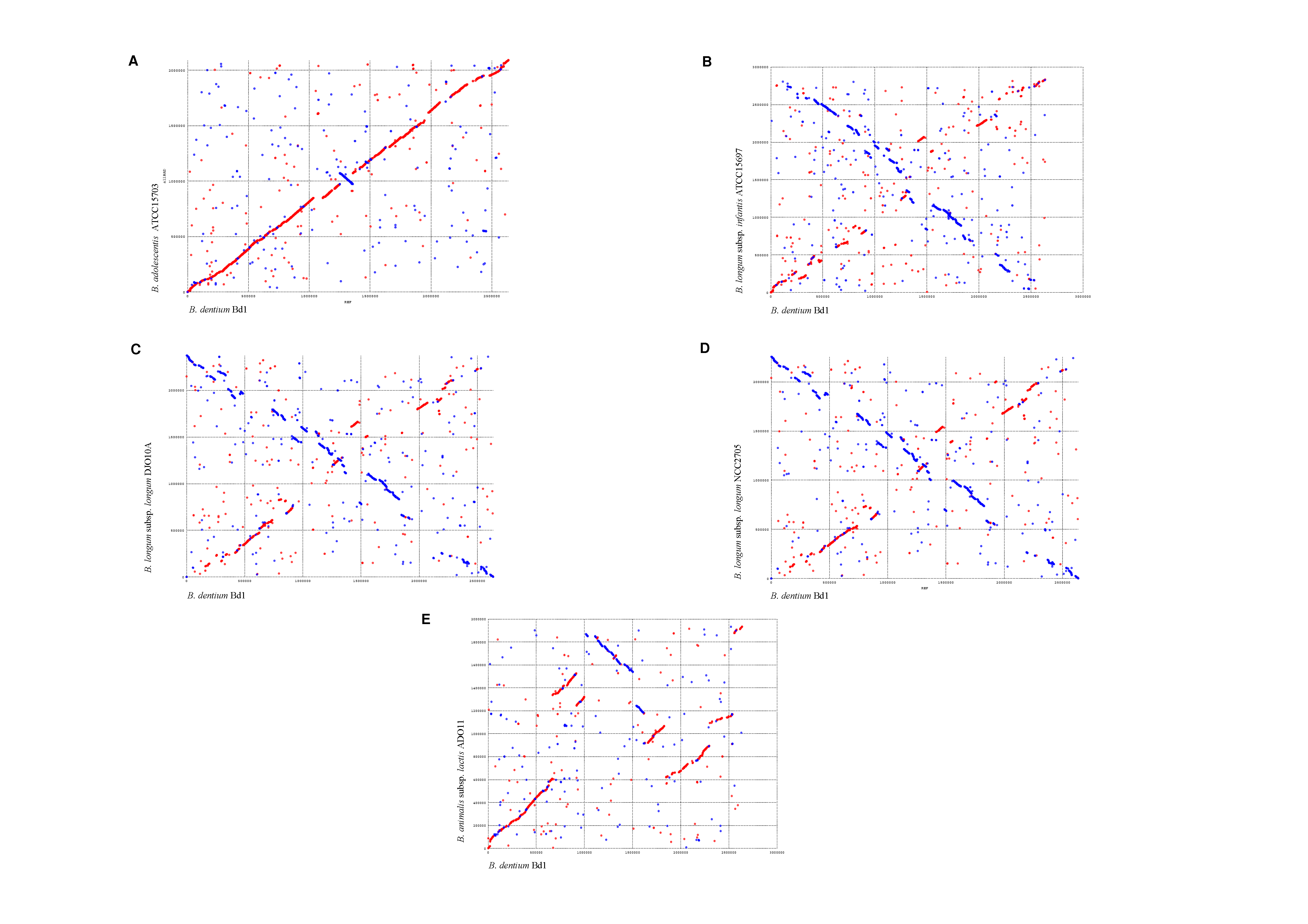

Supplement: Figure S2 — Genome colinearity of B. dentium Bd1 with B. adolescentis ATCC15703 (A), B. longum subsp. infantis ATCC15678 (B), B. longum subsp. longum DJO10A (C), B. longum subsp. longum NCC2705 (D), and B. animalis subsp. lactis ADO11 (E). Each dot matrix was calculated using MUMmer. The comparison window was 50 bp and the stringency was 30 bp. (1.80 MB TIF) [file pgen.1000785.s002.tif]

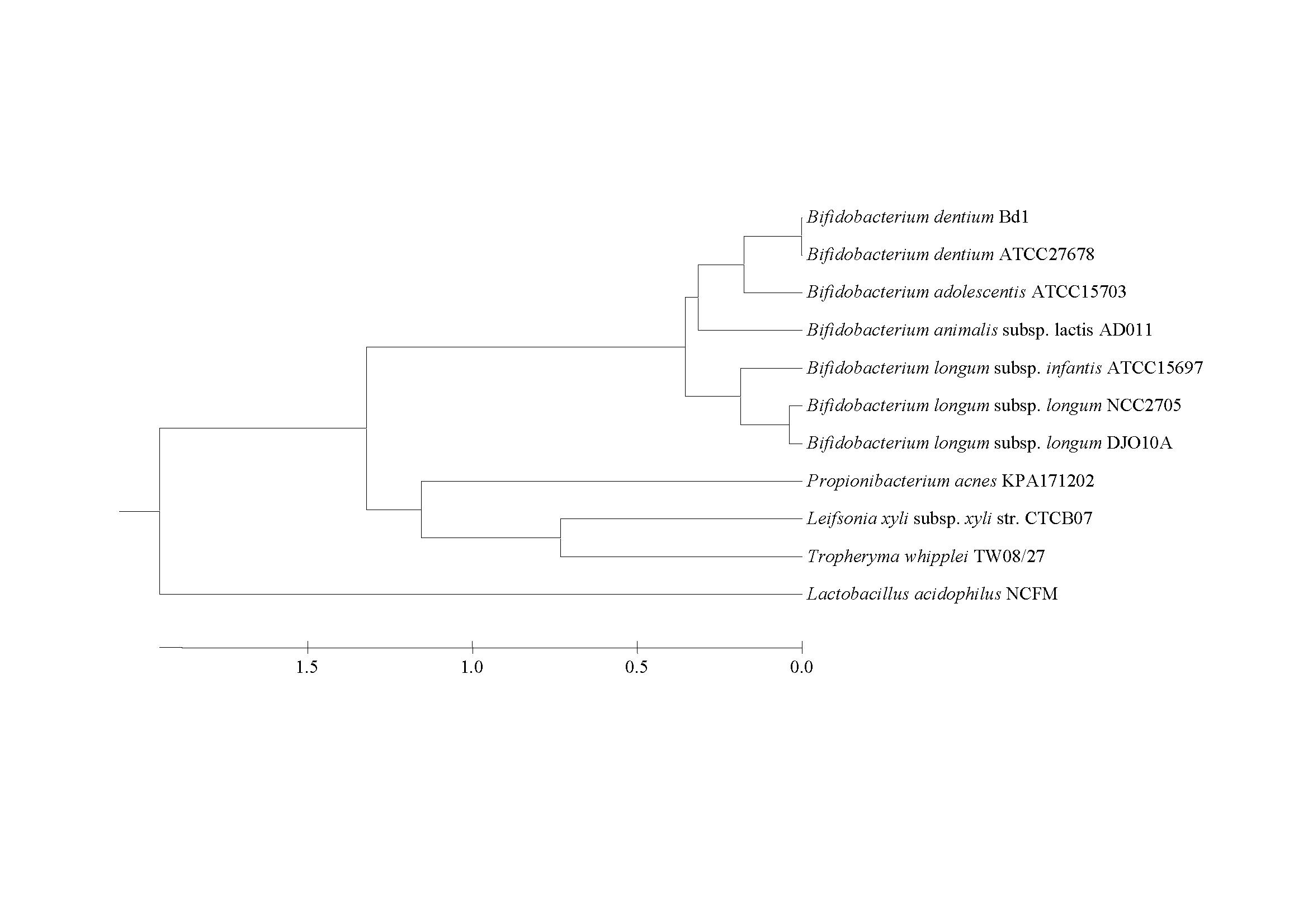

Supplement: Figure S3 — Phylogenetic supertree based on the sequences of Actinobacteria core proteins, using SplitsTree. (0.29 MB TIF) [file pgen.1000785.s003.tif]

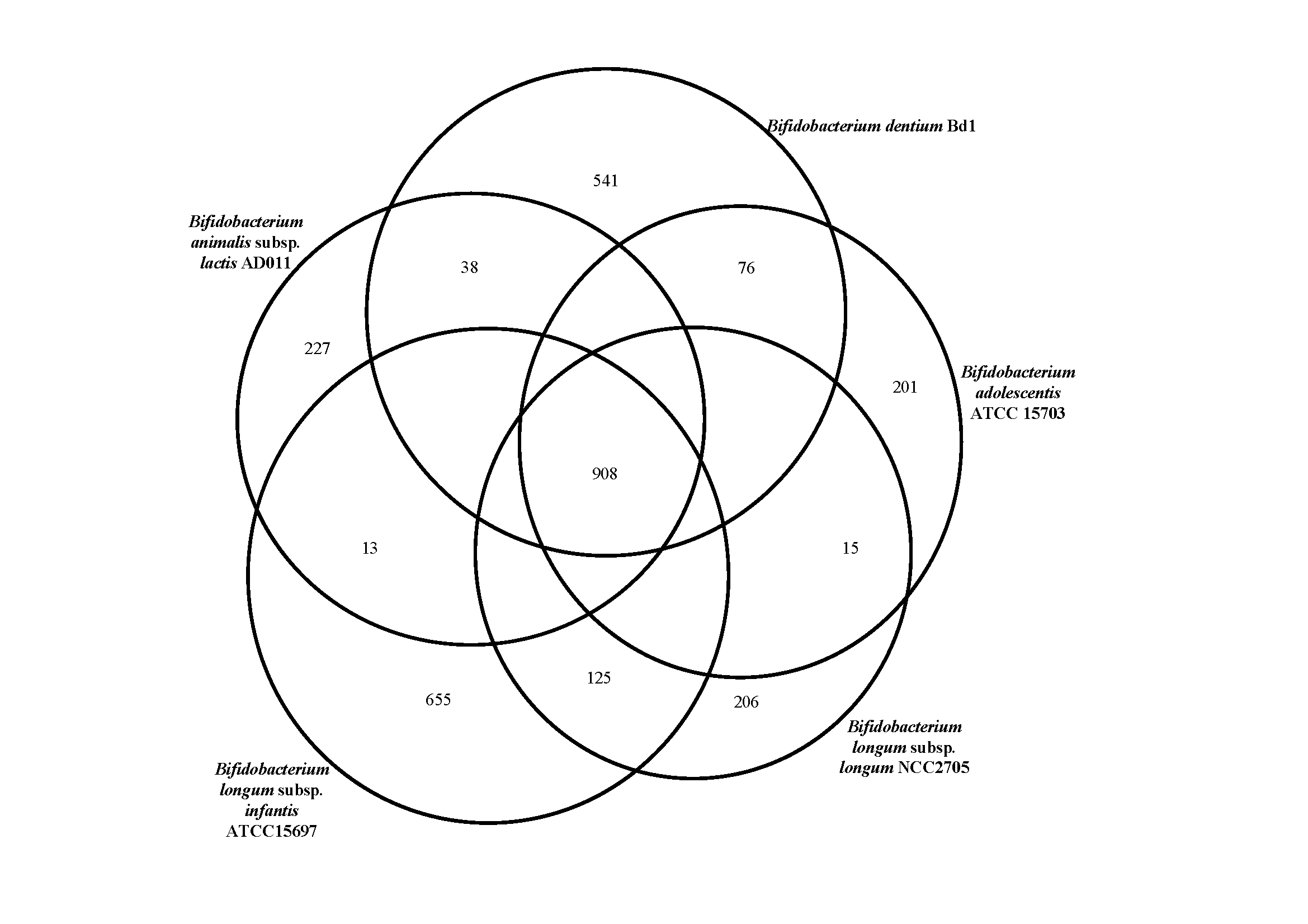

Supplement: Figure S4 — Venn diagram of homologs shared between sequenced bifidobacterial genomes. Circle sizes are proportional to members contained in each set. (0.31 MB TIF) [file pgen.1000785.s004.tif]

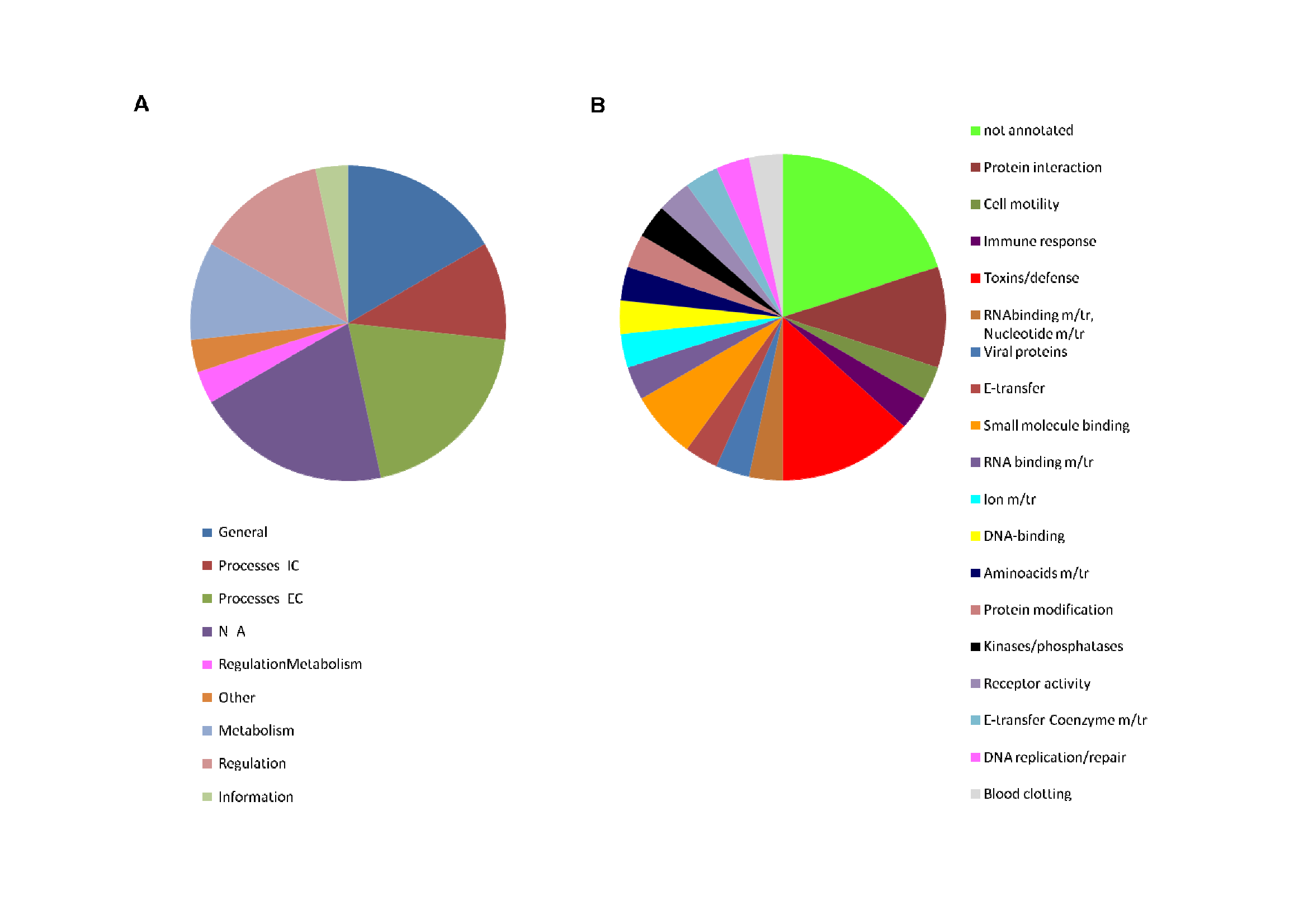

Supplement: Figure S5 — Analysis of the proteins encoded by the genome of B. dentium Bd1 according to the distribution of functions in terms of SCOP Domain Superfamilies. (A) shows a pie chart displaying the proportion of proteins encoded by the genome of B. dentium Bd1 classified according to general functional categories, while (B) shows a pie chart distribution of the proteins encoded by the genome of B. dentium Bd1 when classified according to more detailed functional categories. (0.47 MB TIF) [file pgen.1000785.s005.tif]

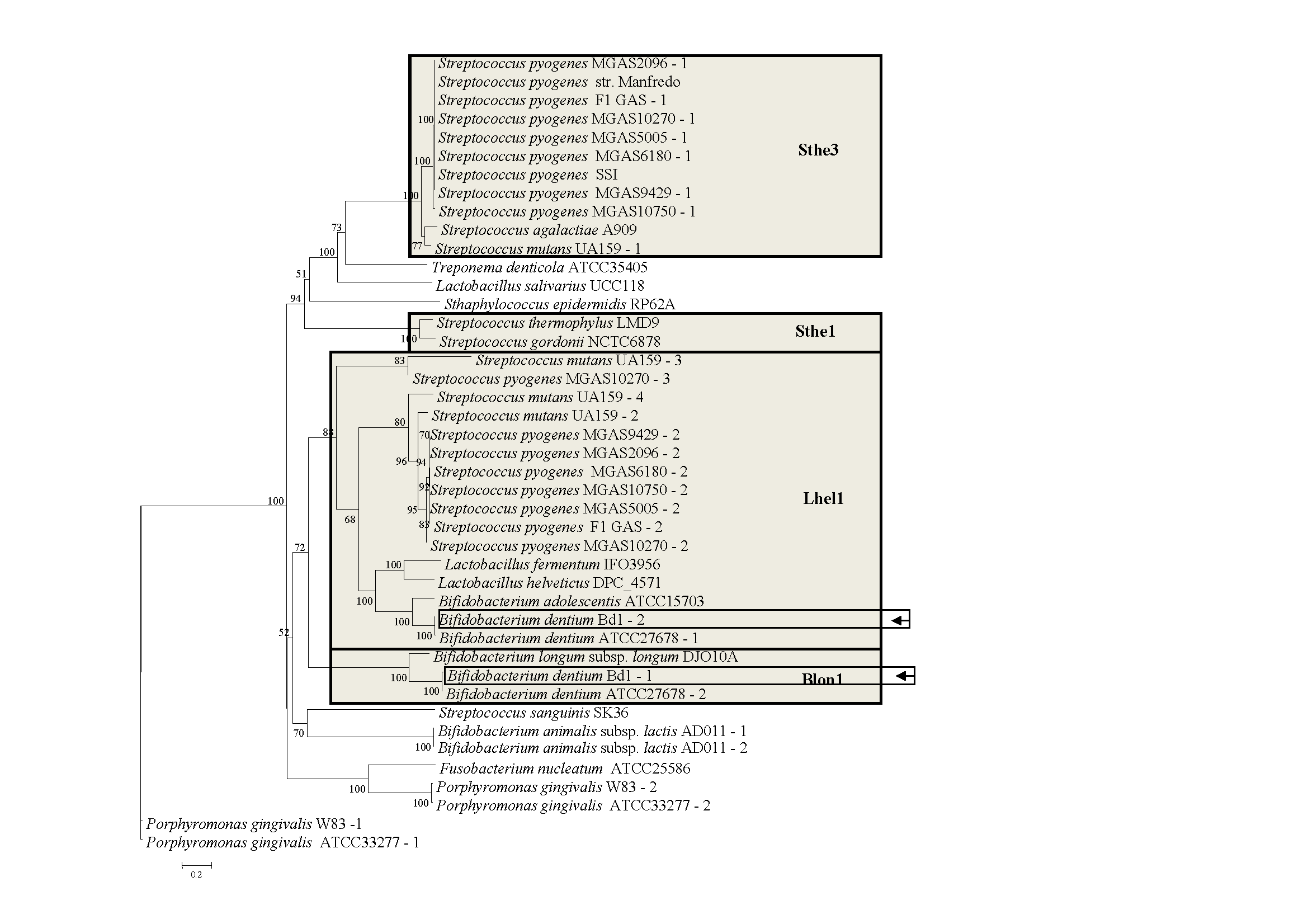

Supplement: Figure S6 — Comparative analysis of Cas1 protein sequences. CRISPR repeat families are indicated within the shaded boxes. Bootstrap values are indicated at the nodes for a total of 1,000 replicates. The arrows pointed the Cas protein in the two CRISPR loci of B. dentium Bd1. Bootstrap values above 40 are shown. (0.39 MB TIF) [file pgen.1000785.s006.tif]

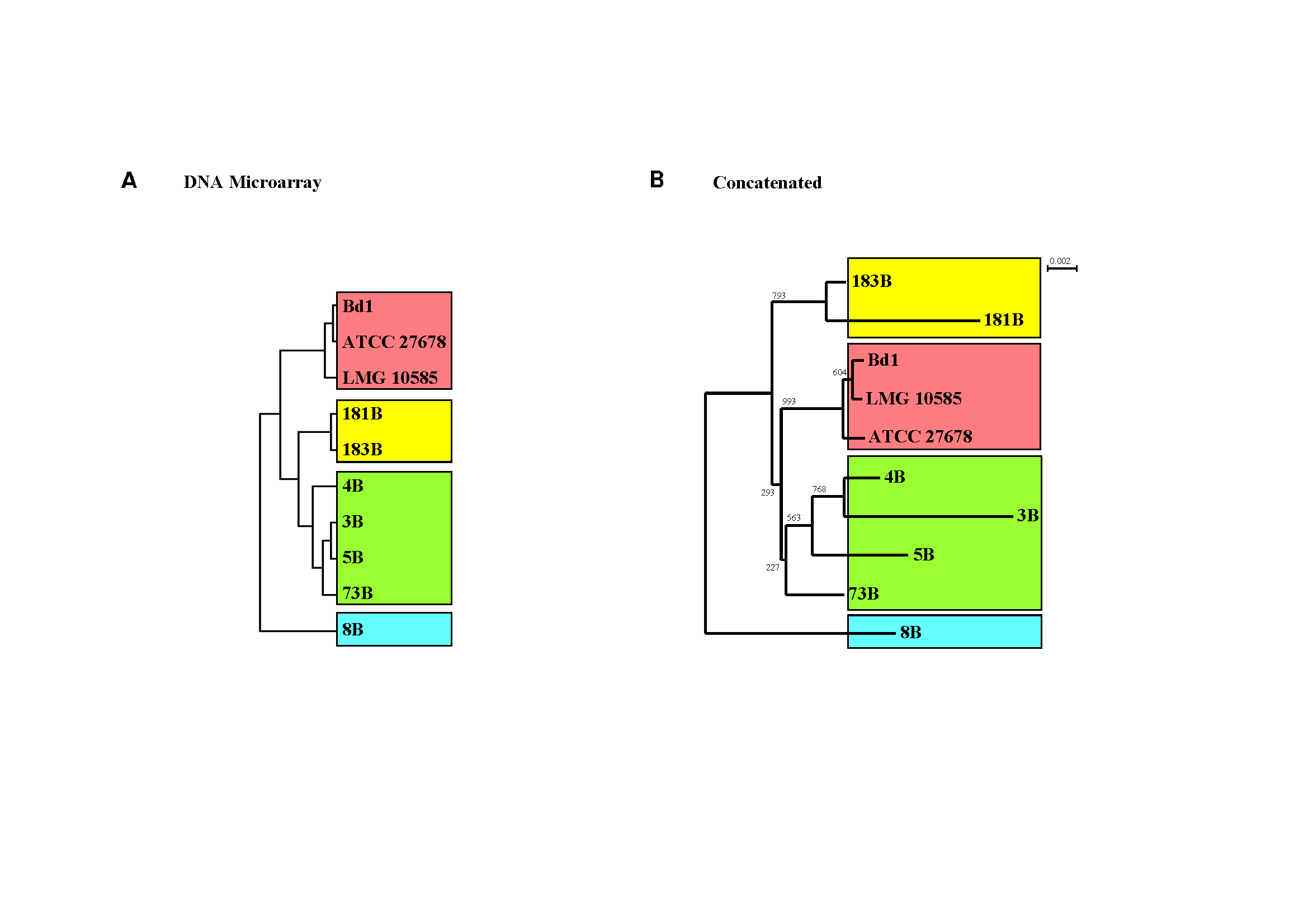

Supplement: Figure S7 — Polyphasic analysis of the genetic diversity in the B. dentium species using the CGH clustering data and a phylogenetic tree of the ten B. dentium strains computed from the concatenation of clpC, dnaJ1, rpoC, and xfp gene sequences by the neighbour-joining method and Kimura's two parameter model as the substitution model. In each tree, the strain is indicated at the right end of the branch, the colour typing indicates the different ecological origin, i.e., red from dental caries, green from saliva of patients with caries, blue from saliva of healthy patients and yellow from fecal samples. The numbers at the nodes relate to the bootstrap probabilities. The different clusters are boxed. (0.34 MB TIF) [file pgen.1000785.s007.tif]

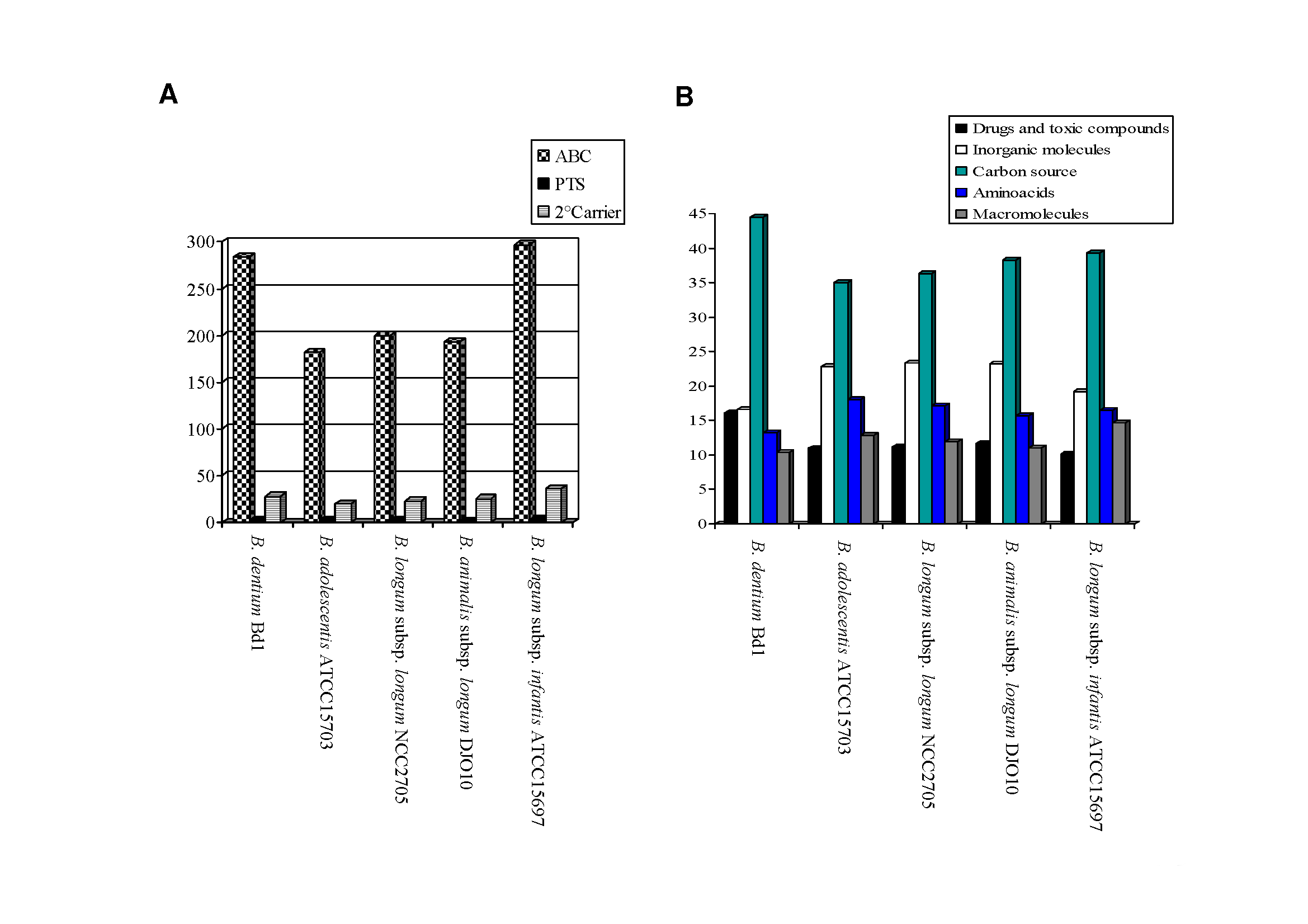

Supplement: Figure S8 — Predicted transport capabilities of B. dentium Bd1 compared to other bifidobacteria (A). Predicted compounds transported by the sequenced bifidobacteria (B). (0.43 MB TIF) [file pgen.1000785.s008.tif]

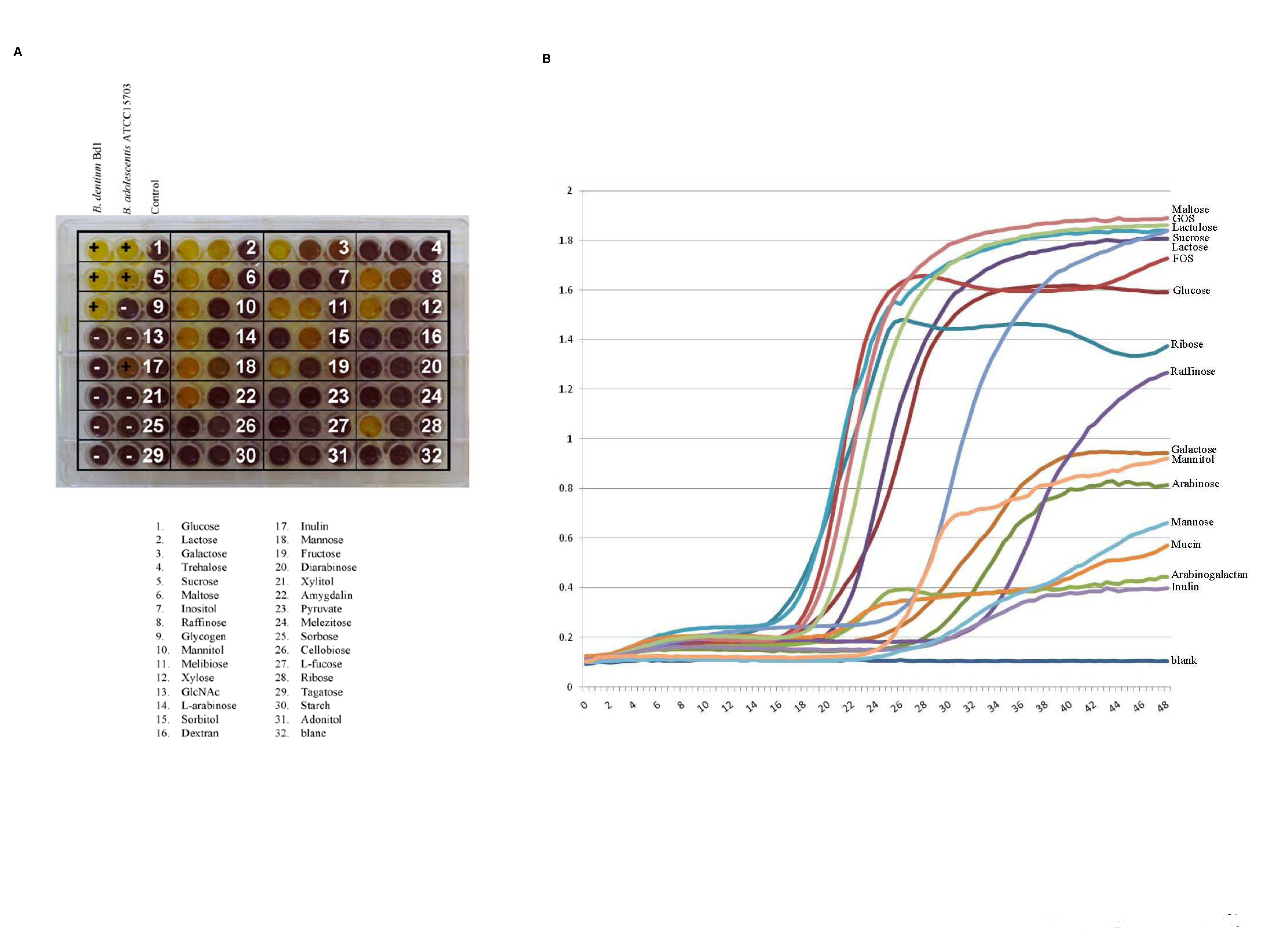

Supplement: Figure S9 — Carbohydrate metabolizing capabilities of B. dentium Bd1. (A) shows the sugar fermentation profiles of B. dentium Bd1 and B. adolescentis ATCC15703 strains, respectively. Carbohydrates used are indicated. + indicates acid production; − indicates absence of acid production. (B) displays the growth curves of B. dentium Bd1 on different carbohydrates as their sole carbon source. The carbohydrates used are indicated next to each curve. (6.37 MB TIF) [file pgen.1000785.s009.tif]

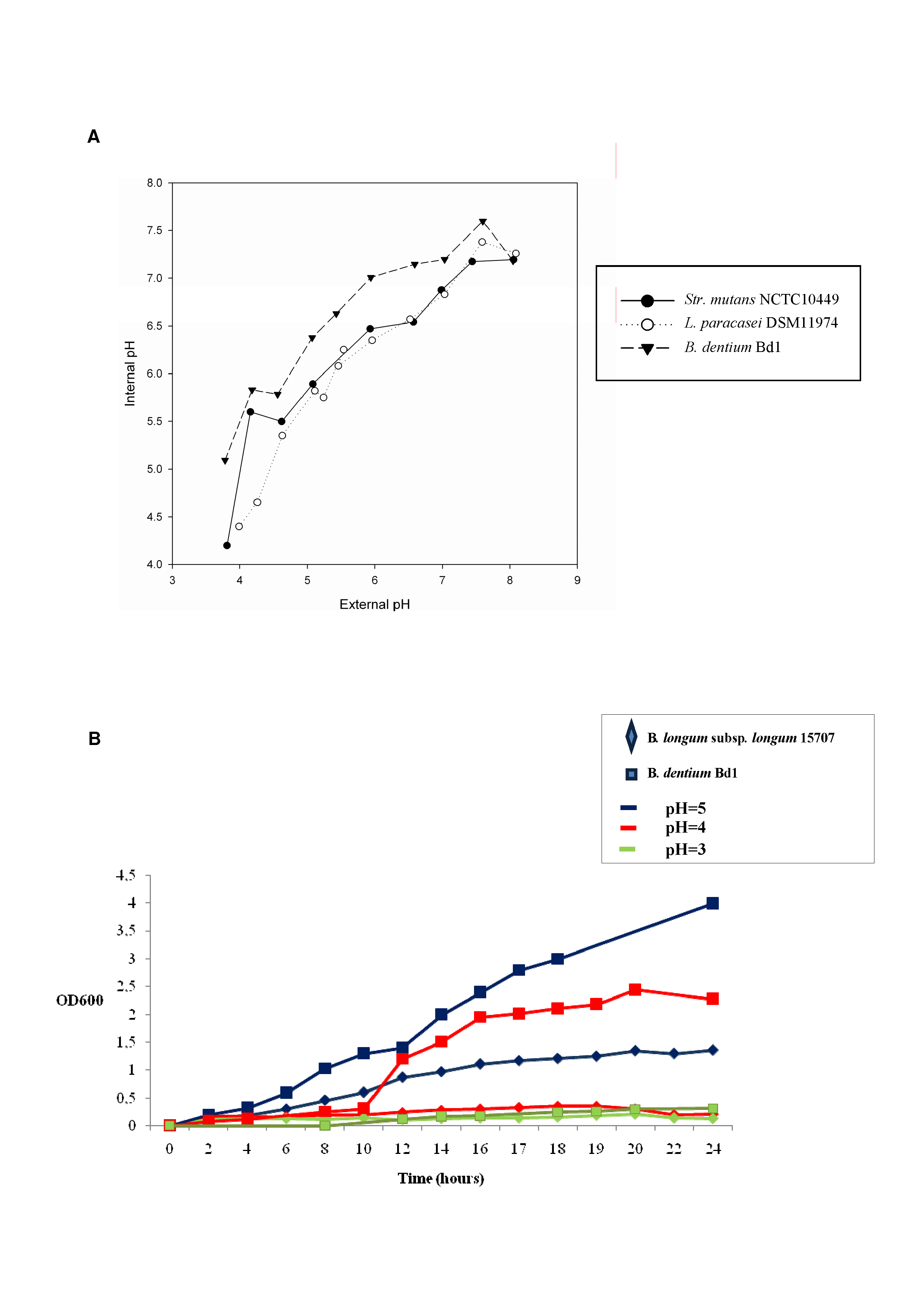

Supplement: Figure S10 — Ecological adaptability of B. dentium Bd1 to acidic environments. (A) indicates the intracellular pH of B. dentium Bd1, S. mutans UA159, and Lb. paracasei subsp. paracasei ATCC11974 at various extracellular pH values. The data obtained from three independent experiments were plotted. In (B), growth of B. dentium Bd1 and B. longum subsp. longum ATCC 15707 cultures maintained at different acidic conditions were monitored over 24 hours. The colour of the line indicates the pH value of the medium used: red, pH 5; blue, pH4; green, pH 3. (0.90 MB TIF) [file pgen.1000785.s010.tif]
