# Supplementary material for: The Bifidobacterium dentium Bd1 Genome Sequence Reflects Its Genetic Adaptation to the Human Oral Cavity
Source: PLoS Genet. 2009 Dec 24;5(12):e1000785. doi: 10.1371/journal.pgen.1000785 (PMC2788695; doi:10.1371/journal.pgen.1000785)
Supplement: Table S1 — Number of ABC exporter and importer system according to substrate type present in the bifidobacterial genome sequenced so date. (0.05 MB DOC) [file pgen.1000785.s011.doc]

| **Category** | **Bd1** | **BIFDEN** | **BAD** | **BL** | **BLD** | **Blon** |
| --- | --- | --- | --- | --- | --- | --- |
| **1)Efflux** | **14** | **13** | **15** | **14** | **13** | **20** |
| Drugs | 9 | 8 | 8 | 7 | 7 | 7 |
| Lipids | 3 | 3 | 3 | 5 | 3 | 3 |
| Peptides | 0 | 0 | 0 | 0 | 1 | 1 |
| Complex carbohydrates | 2 | 2 | 2 | 2 | 2 | 2 |
| Proteins | 0 | 0 | 2 | 0 | 1 | 2 |
| Inorganic cations | 0 | 0 | 0 | 0 | 0 | 6 |
| **2)Uptake** | **284** | **277** | **182** | **200** | **193** | **299** |
| Sugars | 167 | 164 | 85 | 106 | 106 | 145 |
| Aminoacids | 32 | 32 | 30 | 21 | 21 | 33 |
| Inorganic cations | 25 | 24 | 17 | 22 | 18 | 31 |
| Peptides | 17 | 16 | 16 | 20 | 17 | 33 |
| Organic cations | 43 | 41 | 34 | 31 | 31 | 57 |

Bd1: *B. dentium* Bd1; BIFDEN: *B. dentium* ATCCATCC27678; BAD: *B. adolescentis* ATCC15703; BL: *B. longum* subsp. *longum* NCC2705; BLD: *B. longum* subsp. *longum* DJO10A; Blon: *B. longum* subsp. *infantis* ATCC15697
