# Supplementary material for: The Bifidobacterium dentium Bd1 Genome Sequence Reflects Its Genetic Adaptation to the Human Oral Cavity
Source: PLoS Genet. 2009 Dec 24;5(12):e1000785. doi: 10.1371/journal.pgen.1000785 (PMC2788695; doi:10.1371/journal.pgen.1000785)
Supplement: Table S2 — Selected genes upregulated/downregulated upon acidic stress. (0.07 MB DOC) [file pgen.1000785.s012.doc]

| **Locus tag** | **Predicted enzyme function** | **Change (fold)^a^** | **Change (fold)^b^** |
| --- | --- | --- | --- |
|  |  |  |  |
| BDP_0018 | Conserved hypothetical protein | 5 (3.05E-13) | 15 (6.00E-15) |
| BDP_0046 | pip1 hypothetical membrane spanning protein | 11,5 (2.66E-11) | 15,8 (6.32E-12) |
| BDP_0048 | pip2 hypothetical membrane protein pip | 7,2 (7.88E-13) | 14,5 (1.11E-15) |
| BDP_0057 | Serine protease | 58,5 (5.48E-14) | 47,8 (3.75E-12) |
| BDP_0064 | Zn-dependent protease | 15 (2.57E-12) | 7,9 (3.37E-11) |
| BDP_0118 | ilvC ketol-acid reductoisomerase | 7,4 (1.02E-06) | 10 (3.15E-07) |
| BDP_0136 | LacI-type transcriptional regulator | 10,6 (2.95E-13) | 8,7 (8.75E-12) |
| BDP_0141 | Hypothetical protein | 13,3 (1.29E-12) | 21,2 (2.16E-14) |
| BDP_0142 | Sortase | 6,7 (3.36E-11) | 10,1 (3.82E-13) |
| BDP_0179 | Possible adhesin | 65,2 (0) | 118,7 (0) |
| BDP_0180 | Hypothetical protein | 16,7 (8.11E-07) | 36,6 (8.75E-08) |
| BDP_0309 | Aspartate ammonia-lyase | 12,7 (9.20E-14) | 13,3 (2.82E-13) |
| BDP_0339 | ATP-binding protein of ABC transporter system | 20,4 (5.34E-11) | 6,6 (1.28E-09) |
| BDP_0340 | ATP-binding protein of ABC transporter system | 14,7 (3.15E-10) | 5,8 (4.11E-07) |
| BDP_0501 | Narrowly conserved hypothetical membrane spanning protein | 13,3 (0) | 7,4 (4.44E-15) |
| BDP_0721 | ImpB/MucB/SamB family protein involved in DNA repair | 12,6 (1.03E-12) | 15,3 (1.55E-12) |
| BDP_0816 | ATP-binding protein of ABC transporter system | 47,6 (1.21E-09) | 11,4 (1.24E-07) |
| BDP_0817 | Permease protein of ABC transporter system | 39,1 (4.44E-16) | 13,7 (2.35E-14) |
| BDP_0846 | Peptidase E | 14,2 (0) | 11,8 (2.22E-16) |
| BDP_0880 | whiB WhiB-type transcription regulator | 9,4 (6.67E-12) | 11 (4.81E-12) |
| BDP_1106 | cbh Conjugated bile salt hydrolase | 49,9 (3.26E-13) | 96,5 (9.78E-14) |
| BDP_1119 | Malate dehydrogenase | 6,3 (3.33E-15) | 17,8 (0) |
| BDP_1150 | Conserved hypothetical protein | 8,6 (1.46E-11) | 10,4 (3.15E-11) |
| BDP_1274 | Transcriptional regulator, GntR family | 3,2 (2.24E-07) | 12,3 (1.78E-10) |
| BDP_1295 | argJ argJ Glutamate N-acetyltransferase/Amino-acid acetyltransferase | 9,5 (4.59E-12) | 12,1 (9.35E-12) |
| BDP_1301 | Permease protein of ABC transporter system | 40 (7.14E-14) | 42,8 (2.60E-13) |
| BDP_1302 | ATP-binding protein of ABC transporter system | 64,9 (8.66E-15) | 67,3 (1.25E-14) |
| BDP_1423 | Transcriptional regulator 1600078:1600578 reverse | 5,7 (2.78E-12) | 10,3 (4.67E-13) |
| BDP_1493 | oppA2 oppA2 Oligopeptide-binding protein oppA | 6,9 (9.68E-13) | 11,3 (1.29E-13) |
| BDP_1555 | lysM LysM domain protein | 10,2 (6.95E-14) | 9,5 (1.21E-13) |
| BDP_1620 | Hypothetical protein | 13,4 (6.75E-13) | 9,5 (2.44E-12) |
| BDP_1749 | gadB glutamate decarboxylase | 27,6 (0) | 90,5 (0) |
| BDP_1750 | Glutamate:g-aminobutyrate antiporter | 14,1 (1.04E-10) | 51 (4.02E-11) |
| BDP_1759 | Heavy metal translocating P-type ATPase | 4,7 (1.25E-07) | 14,7 (1.02E-06) |
| BDP_1963 | frc formyl-coenzyme A transferase | 31,9 (6.64E-10) | 117 (7.00E-12) |
| BDP_1965 | Permease similar to auxin efflux carriers | 8,8 (1.31E-05) | 30,2 (2.83E-07) |
| BDP_1966 | oxc oxalyl-CoA decarboxylase | 39,8 (5.56E-11) | 135,1 (5.44E-15) |
| BDP_1970 | Conserved hypothetical membrane spanning protein | 4,7 (3.78E-12) | 13,5 (2.33E-15) |
| BDP_2004 | Conserved hypothetical protein | 16,6 (0) | 41,7 (0) |
| BDP_2141 | adh2 adh2 Aldehyde-alcohol dehydrogenase | 5,7 (1.31E-07) | 11,5 (1.45E-08) |
| BDP_2142 | argD argD Acetylornithine aminotransferase | 11,3 (5.14E-09) | 33,8 (3.26E-10) |
| BDP_2177 | hspR Heat shock regulatory protein | 14,9 (6.14E-13) | 3,1 (2.08E-08) |
| BDP_2178 | DnaJ1 chaperone protein | 13,3 (1.21E-11) | 3,2 (6.65E-08) |
| BDP_2179 | GrpE protein | 27,6 (6.67E-12) | 3,8 (1.46E-09) |
| BDP_2180 | DnaK Chaperone protein dnaK | 24,8 (1.61E-14) | 4,3 (1.29E-10) |

^a^ Genes upregulated and downregulated of *B. dentium* cultures exposed 30 min at pH of 4.

^b^ Genes upregulated and downregulated of *B. dentium* cultures exposed 2 hours at pH of 4.

^a,b^ Values in parenthesis indicate the p-value.
