# Supplementary material for: The Bifidobacterium dentium Bd1 Genome Sequence Reflects Its Genetic Adaptation to the Human Oral Cavity
Source: PLoS Genet. 2009 Dec 24;5(12):e1000785. doi: 10.1371/journal.pgen.1000785 (PMC2788695; doi:10.1371/journal.pgen.1000785)
Supplement: Table S3 — Selected genes differentially expressed upon B. dentium Bd1 growth in different sugar-based media relative to growth in glucose. (0.09 MB DOC) [file pgen.1000785.s013.doc]

| **Locus tag** | **Gene** | **Predictive enzyme function** | **Ribose ^a^** | **Maltose ^b^** | **Mannitol ^c^** | **Amygdalin ^d^** |
| --- | --- | --- | --- | --- | --- | --- |
| BDP_0010 |  | H+/gluconate symporter or related permease | -5,2 (6.00E-15) | -41,8 (6.58E-11) | -22,1 (3.21E-12) | -12,5 (1.49E-13) |
| BDP_0011 |  | Hexulose-6-phosphate isomerase | -5,5 (3.62E-12) | -28,4 (9.82E-08) | -25,3 (4.73E-11) | -10,1 (3.32E-11) |
| BDP_0012 |  | Sugar kinase | -4,8 (9.73E-11) | -25,5 (1.49E-08) | -15,2 (7.84E-09) | -7,8 (4.95E-08) |
| BDP_0013 |  | 3-hexulose-6-phosphate synthase | -4,3 (6.86E-13) | -15,7 (4.82E-11) | -11,3 (1.02E-08) | -7,7 (6.22E-12) |
| BDP_0014 |  | 3-hexulose-6-phosphate isomerase | -3,2 (2.00E-11) | -7,2 (5.56E-09) | -4,1 (8.77E-08) | -4,5 (1.14E-09) |
|  |  |  |  |  |  |  |
| BDP_0170 | *rbs*R1 | LacI-type transcriptional regulator | 32,8 (6.77E-15) | 1,7 (5,47E-03) | 1,4 (1,21E-02) | 1 (5,65E-01) |
| BDP_0171 | *rbs*R2 | LacI-type transcriptional regulator | 91,2 (0) | -1,3 (2,38E-02) | 1,2 (1,53E-01) | -1,6 (8.45E-07) |
| BDP_0172 | *rbs*A | ABC-type ribose transporter ATPase component | 106,8 (0) | -1 (5,78E-01) | -6 (3.02E-07) | -3,6 (3.78E-12) |
| BDP_0173 | *rbs*C | ABC-type ribose transporter permease component | 43,4 (4.36E-12) | -1,1 (2,28E-01) | -4,7 (3,52E-03) | -3,5 (1.26E-06) |
| BDP_0174 | *rbs*B | ABC-type ribose transporter sugar-binding component | 97,9 (3.33E-16) | -1,1 (2,70E-01) | -5,7 (9.05E-07) | -4,3 (2.18E-07) |
| BDP_0175 | *rbs*D | D-ribose pyranase | 92 (2.55E-15) | -1,3 (8,91E-01) | -4,5 (9.06E-06) | -4 (4.23E-07) |
|  |  |  |  |  |  |  |
| BDP_0326 |  | Oxidoreductase | 2,4 (3.27E-07) | 1,1 (5,54E-02) | 2,9 (4.00E-07) | 23,7 (6.13E-07) |
|  |  |  |  |  |  |  |
| BDP_0423 |  | Mannitol dehydrogenase | 1 (3,93E-03) | -1,7 (1,58E-02) | 224,7 (2.45E-13) | -1,4 (8,14E-04) |
| BDP_0424 |  | Mannitol permease, MFS superfamily | 1,1 (4,97E-02) | -2 (1,74E-03) | 106,2 (4.88E-15) | -1,2 (2,34E-02) |
| BDP_0425 |  | Transcriptional regulator, ROK family | 0,9 (1.75E-08) | -1,2 (1,12E-01) | 32,8 (1.89E-15) | 2,4 (1.02E-08) |
|  |  |  |  |  |  |  |
| BDP_0624 | *agl*2 | Alpha-glucosidase | -3,7 (4.27E-09) | 38,6 (6.41E-12) | -1,4 (4,17E-02) | -1,8 (3,08E-04) |
| BDP_0625 | *scr*T | ScrT Sucrose transporter | -2,7 (1.07E-05) | 38,6 (1.30E-12) | -1,3 (4,89E-02) | -1,7 (3,18E-04) |
|  |  |  |  |  |  |  |
| BDP_1671 | *bgl*2 | Beta-glucosidase | -1,3 (1.51E-05) | 1 (8.03E-05) | -2,8 (1.85E-05) | 15,8 (7.99E-15) |
|  |  |  |  |  |  |  |
| BDP_2026 | *iun*H | Inosine-uridine preferring nucleoside hydrolase | 13,1 (5.05E-12) | 1,7 (2,07E-01) | 1,2 (3,02E-02) | 1,1 (3,94E-01) |
| BDP_2025 |  | Transporter, MFS superfamily | 9,9 (3.74E-11) | -1,4 (7.83E-05) | -1,2 (7,22E-02) | -1,3 (6,08E-03) |
| BDP_2024 | *rbs*K | Ribokinase | 18,1 (1.39E-14) | -2 (5.04E-05) | -1,3 (4,99E-02) | -1,5 (2,78E-04) |
|  |  |  |  |  |  |  |
| BDP_2070 |  | ABC-type glucose transporter sugar-binding component | -60,3 (5.44E-15) | -127,5 (2.73E-13) | -205,6 (0) | -196,2 (3.25E-13) |
| BDP_2069 |  | ABC-type glucose transporter ATPase component | -53,1 (0) | -102,3 (0) | -222,6 (0) | -177,3 (0) |
| BDP_2068 |  | ABC-type glucose transporter permease component | -15,2 (1.88E-10) | -22,5 (1.40E-08) | -75,5 (5.43E-13) | -25,4 (1.15E-09) |
| BDP_2067 |  | Conserved hypothetical protein | -17,4 (0) | -25,9 (9.99E-16) | -85,3 (0) | -33 (0) |
|  |  |  |  |  |  |  |
| BDP_2259 | *bgl*5 | Beta-glucosidase | -1,3 (8.60E-05) | -2,6 (1.58E-06) | -1,8(4,21E-04) | 53,2 (1.52E-13) |
| BDP_2253 | *bgl*4 | Beta-glucosidase | 1,1 (8,90E-02) | -2,9 (1,40E-04) | 2,1 (6,36E-04) | 21,1 (3.02E-13) |
| BDP_2252 |  | Putative sugar transporter | 1 (9,36E-01) | -1,1 (1.12E-06) | -1,5 (5,97E-04) | 10,5 (1.11E-15) |

^a, b, c, d^ Genes upregulated/downregulated in *B. dentiu*m Bd1 cells grown in MRS-plus ribose or maltose or mannitol or amygdalin vs Bd1 cells grown in MRS supplemented with glucose. Values in parenthesis indicate the p-value
