# Supplementary material for: The Bifidobacterium dentium Bd1 Genome Sequence Reflects Its Genetic Adaptation to the Human Oral Cavity
Source: PLoS Genet. 2009 Dec 24;5(12):e1000785. doi: 10.1371/journal.pgen.1000785 (PMC2788695; doi:10.1371/journal.pgen.1000785)
Supplement: Text S1 — Predicted interactions between B. dentium Bd1 and host, genetic regions of oral pathogens present in the B. dentium Bd1 genome and detailed description of the experimental procedures. (0.13 MB DOC) [file pgen.1000785.s014.doc]

**TextS1.**

**Predicted interactions between *B. dentium* Bd1 and host.** Pathogens have evolved various strategies to intercept, mimic and usurp cellular processes of their hosts in order to successfully acquire nutrients and evade immune defences [1,2]. Direct protein-protein interactions (PPIs) between pathogen and host proteins play a key role in such strategies [3]. Identifying PPIs between *B. dentium* Bd1 and its host will therefore provide valuable information on the pathogenic activities of this microbe. With the assistance of the InParanoid algorithm [4] in detecting orthologs, the interolog approach was used to infer the interactome between *B. dentium* Bd1 and its human host, based on the experimentally verified PPI data from *E. coli*, *Campylobacter jejuni, Helicobacter pylori,* *Saccharomyces cerevisiae*, *Caenorhabditis elegans*, *Drosophila melanogaster* and *Homo sapien*s. To find orthologs for a query sequence, InParanoid firstly identifies all the potential orthologs in targeting organism by pair-wise similarity searching and then clusters these potential orthologs into groups of likely co-orthologs. In our analyses, we only selected the main orthologs to predict the PPIs. Considering only PPIs of the extracellular proteins, we obtained 528 PPIs among 408 human and 95 pathogen proteins. Furthermore, when the obtained *B. dentium* Bd1-human interactome was compared to that of another, intestinal bifidobacterial species (*B. longum* subsp. *longum* NCC2705), 150 human proteins were predicted to uniquely interact with *B. dentium* Bd1. Such proteins involved three different GO categories, i.e., protein metabolic process, signal transduction and cell communication. Furthermore, 95 *B. dentium* Bd1 proteins were shown to specifically interact with the human host, and mainly involve proteins required for transport, primary metabolic and biosynthetic processes. Notably, a human protein (NP_002735.3), which is involved in inflammatory response [5,6], was predicted to interact with a cysteine, histidine-dependent amidohydrolase/peptidase of *B. dentium* Bd1 (BDP_0643). Interestingly, none of the annotated functions of human proteins that were predicted to interact with the proteome of the intestinal commensal *B. longum* subsp. *longum* NCC2705 are linked to the inflammatory response.

**Genetic regions of oral pathogens present in the *B. dentium* Bd1 genome.** In order to further investigate the apparent genetic adaptation of *B. dentium* Bd1 to the oral cavity, we expanded our analyses to the *in silico* identification of chromosomal regions of *B. dentium* that have homologous regions in the genomes of oral pathogens and that are absent from those of intestinal bifidobacteria. Genomes of a large number of oral pathogens including both strictly and opportunistic pathogenic bacteria, belonging to a wide range of taxonomic groups have been sequenced: *S. mutans* UA159, *Streptococcus mitis* NCTC12261, *S. gordonii* NCTC6878, *Streptococcus sanguinis,* eleven *Streptococcus pyogenes* strains (e.g., M1 GAS, MGAS10270, MGAS10394, MGAS10750, MGAS2096, MGAS315, MGAS5005, MGAS6180, MGAS8232, MGAS9429, SSI-1, str. Manfredo), *F. nucleatum* ATCC25586, *T. denticola* ATCC35405, *Porphyromonas gingivalis* W83 and *Porphyromonas gingivalis* ATCC33277, *Actinomyces odontolyticus* ATCC 17982 [7,8]. Remarkably, when the genome sequence of *B. dentium* Bd1 was compared to a genome database representing these oral pathogenic strains mentioned above (oral pathogen pangenome), a large number (around 1390) of significant BLAST matches (with an e-value lower than 10^-4^ and identity greater than 30% at amino acid level) were returned. These do not represent extended contiguous *B. dentium* Bd1 DNA regions, but rather discrete genes that were distributed across the chromosome. Notably, some of these include genes encoding cell-membrane proteins or cell-surface structures that could have a role in cell adhesion, which have homologs on the genomes of *T. denticola*, *S. mutans*, *S. sanguinis* and *F. nucleatum*. Moreover, other Bd1 genes with homologs in the oral pathogen pangenome include those encoding glutamine/glutamate transporters or coding for enzymes involved in sugar breakdown. Interestingly, a clear example of horizontally acquired DNA region from an oral pathogen is represented by the DNA fragment of Bd1 genome encompassing five ORFs (from BDP_1391 to BDP_1394), flanked by two IS elements, displaying similarities with cytosolic proteins encoded by the Gram negative bacterium *F. nucleatum*.

**Bacterial strains, growth conditions and chromosomal DNA extraction**. *B. dentium* Bd1 was acquired from the Belgian Co-ordinated Collections of Microorganisms (BCMM) as LMG 11045, which is equivalent to ATCC27534. Cultures were grown anaerobically in MRS (Sharlau, Barcelona, Spain) supplemented with 0.05% L-cysteine-HCl and incubated at 37°C for 16 h. Bacterial DNA was extracted as described previously [9] and submitted to further purification using the Genomic DNA Purification Kit (Qiagen, UK) for Comparative genomic hybridizations.

**Carbohydrate growth assay**. Fermentation of carbohydrates was assayed in basic medium [10] supplemented with 2% of a particular carbohydrate. Inoculum was about 10^9^ CFU/ml. The assay does not measure growth but production of acid, which was detected with bromochresol purple.

Cell growth on semi synthetic MRS medium supplemented with 2% (wt/vol) of a particular sugar was monitored by optical density at 600 nm using a VersaMax plate reader (Molecular Devices). The plate reader was run in discontinuous mode, with absorbance readings performed at 60 min intervals, and preceded by 30 sec shaking at medium speed. Cultures were grown in biologically independent duplicates and the resulting growth data were expressed as the mean of these replicates.

**Genome sequencing and assembly.** Two whole genome shotgun libraries were constructed for end sequencing. A 3 kb insert library was constructed by randomly shearing 5 μg of chromosomal DNA by using a Hydroshear (GeneMachines) and end repairing by T4 DNA polymerase and Klenow fragment (New England Biolabs). A fosmid library was constructed using the CopyControl^TM^ Fosmid Production Kit (Epicentre). DNA was sheared and fragment size selected on an agarose pulsed-field gel electrophoresis, excised and purified before ligation in the pCCqFos vector. The ligated vector was packaged using a MaxPlax^TM^ Lambda Packaging Extract (Epicentre) kit and used to transduce *Escherichia coli* (EP300). DNA for sequencing was produced using Templiphi (GE HeathCare) on aliquots of subclones grown in 384-well plates according to product specifications. Standard cycle sequencing from both ends of the subclones using universal primers was performed with BigDye Terminators (Applied Biosystems) and resolved on ABI Prism 3730xl capillary sequencers. Sequence reads were assembled using Phred, Phrap, RepeatMasker and the Staden package software [11,12,13] into 38 contigs. Gaps were closed using additional sequencing from small-insert clones (1250 sequencing reads), resulting in a single contig (2,636,368 bp). Quality improvement of the genome sequence involved sequencing of over 200 PCR products (1,200 sequencing reads) across the entire genome to insure correct assembly, double stranding and the resolution of any remaining base-conflicts. The genome sequence was finally edited to a Phred confidence value at least 30. Based on the final consensus quality scores, we estimate an overall error rate of <1 error per 10^5^ nucleotides.

**Sequence annotation.** Protein-encoding open reading frames (ORFs) were predicted using a combination of the methods Glimmer [14] and FrameD [15] as well as comparative analysis involving Orpheus [16] and BLASTX [17]. Results of the four gene finder programs were combined manually and a preliminary identification of ORFs was performed on the basis of BLASTP [18] analysis against a non-redundant protein database provided by the National Centre for Biotechnology Information. Artemis [19] was employed to inspect the results of the combined gene finders and its associated BLASTP results, which was used for manual editing in order to check, or if necessary, redefine the start of every predicted coding region, or to remove or add coding regions.

Assignment of protein function to predicted coding regions of *B. dentium* Bd1 genome was performed manually using the ERGO annotation tool [20]. Moreover, The revised gene/protein set was searched against the Swiss-Prot/TrEMBL, PRIAM, protein family (Pfam), TIGRFam, Interpro, KEGG, and COGs databases, in addition to BLASTP vs. NR. From all these results, functional assignments were made. Manual corrections to automated functional assignments were completed on an individual gene-by-gene basis as needed.

**Bioinformatic analyses**. Ribosomal RNA genes were detected on the basis of BLASTN searches and annotated manually. Transfer RNA genes were identified using tRNAscan-SE [21]. Insertion sequence elements were identified using Repeatfinder [22], and BLAST [18] and annotated manually. IS families were assigned using ISFinder (<http://www-is.biotoul.fr/is.html>). Carbohydrate-active enzymes were identified based on similarity to the carbohydrate-active enzyme (CAZy) database entries [23] and transporter classification was performed according to the TC-DB scheme [24].

Variances in GC content were profiled by the DNA segmentation algorithm hosted at <http://tubic.tju.edu.cn/GC-Profile/> [25], atypical codon usage regions were mapped using the factorial correspondence analysis through the assistance of the GCUA software [26].

Whole genome comparisons of *B. dentium* Bd1 were performed with BLASTN alignments against *B. longum* subsp. *longum* NCC2705 (AE014295), *B. longum* subsp. *longum* DJO10A (CP000605), *B. longum* subsp. *infantis* ATCC15697 (CP001095), *B. adolescentis* ATCC15703 (NZ_BAAD00000000) and *B. animalis* subsp. *lactis* ADO11 (NC_011835). Genome synteny comparisons were performed using the same set of genomes as mentioned above. Whole genomes were compared at the nucleotide level using Dotter [27] and MUMmer programs [14] applying default values.

**CGH Microarray, description, labelling and hybridizations.** CGH analysis was performed with a *B. dentium* Bd1 array. A total of 3778 probes of 35-40 bp in length were designed on 2143 ORFs with a spacing among adjacent probes of at least 700 bp using OligoArray 2.1 software [28]. Oligos were synthesized in triplicate on a 12k CombiMatrix array (CombiMatrix, Mulkiteo, USA). Replicates were distributed on the chip in random, non-adjacent positions. A set of 19 negative control probes designed on phage and plant sequences were also included on the chip. Two μg of purified genomic DNA were labeled with Cy5-ULS using Kreatech ULS array CGH Labeling kit (Kreatech Diagnostics; The Netherlands) according to the supplier’s instructions. Hybridization of labeled DNA to *B. dentium* Bd1 arrays was performed according to CombiMatrix protocols (http://www.combimatrix.com/support_docs.htm).

**Microarray data acquisition and treatment.** Fluorescence scanning was performed on a ScanArray 4000XL confocal laser scanner (Perkin-Elmer). Signal intensities for each spot was determined using Microarray Imager 5.8 software (CombiMatrix, Mulkiteo, USA). Signal background was calculated as the mean of negative controls plus 2 times the standard deviation [29]. A global quantile normalization was performed [30] and log2 ratios among the reference sample (*B. dentium* Bd1) and the other samples analyzed were calculated. The distribution of the log2-transformed ratios was calculated for each hybridization reaction separately. Log2-transformed ratios of each probe were visualized and ranked by position on the *B. dentium* Bd1 genome by a heatmap using TMev 4.0 software (http://www.tm4.org/mev.html). Hierarchical clustering was performed with average linkage and euclidean distance [31] using TMev 4.0 software.

**Multilocus sequence analysis.** The different gene sequences were amplified by PCR using the primers described previously [32], and the PCR products were sequenced on both strands (performed by MWG, Germany). Phylogenetic trees were calculated by the maximum–parsimony and neighbour-joining methods. The reliability of the groups was evaluated by bootstrap with 1000 resamplings.

**Interactomics analyses**. Orthologs of *B. dentium* or proteins in *E. coli*, *C. jejuni, H. pylori, S. cerevisae*, *C. elegans*, *D. melanogaster* and *H. sapiens* were identified through InParanoid with default settings. To find orthologs for a query sequence, InParanoid firstly identifies all the potential orthologs in a targeted organism by pair-wise similarity searching, and then clusters these potential orthologs into groups of likely co-orthologs. Here, we only selected the main orthologs to predict the PPIs.

For a pair of human and bacterium proteins, we predict they can interact if both of them have known interacting orthologs in either *E. coli*, *C. jejuni, H. pylori, S. cerevisae*, *C. elegans* or *D. melanogaster.* In addition, if a bacterial protein has an ortholog encoded by the human genome, we predict that this bacterial protein interacts with its human ortholog’s interacting partners. We identified the secreted proteins with SignalP [33] and the membrane proteins through TMHMM [34]. We excluded bacterial proteins that were predicted to be localized within the cell and thus are unlikely to meet their interacting host protein. The sources of PPI and protein sequence data of model organisms are shown in Table 1. The GO annotations for the human genome were downloaded from HPRD [35], while the GO annotations for the two bacteria were generated through Interproscan [36].

**RNA isolation.** RNA was isolated according to the protocol described previously [37]. The quality of the RNA was checked by analysing the integrity of rRNA molecules by gel electrophoresis.

**Expression microarray**. Reverse transcription reactions contained 10 μg of total RNA, 5 μg of random hexamers, the first strand buffer (75 mM KCl, 50 mM Tris-HCl, 3mM MgCl2), 0.63 mM each of dATP, dCTP, and dGTP, 0’.31 mM dTTP (Invitrogen, Life Technologies, Carlsbad, CA) and 0.31 mM aminoallyl dUTP (Ambion, Austin TX), 5 mM DTT, and 800 U of SuperSript III reverse transcriptase (Invitrogen). The reaction mixture was incubated at 42°C for 2 h. The RNA was hydrolysed by incubation with 0.5M EDTA and 1M NaOH at 65°C for 15 min before purification of the cDNA with GFX columns (GE Healthcare Life Technology). The cDNAs were coupled with monoreactive Cy3 or Cy5 (40 nmol) (Amersham Biosciences, Piscataway, NJ) in the presence of 0.1 M NaHCO3 for 60 min at room temperature. The labelled cDNAs were purified using GFX columns (GE Healthcare Life Technology), combined and vacuum dried. Samples were then suspended in hybridization buffer according to the producer (Agilent Technologies, Santa Clara, CA).

DNA microarrays of *B. dentium* Bd1 consisted of 2114 oligonucleotides spotted on a glass slide (Agilent). Slides were hybridized at 65°C for 17 hours, after which microarray slides were washed following the supplier’s protocol (Agilent).

The hybridized arrays were scanned using an Agilent G2565AA microarray scanner system. Slide data were processed as previously described [38,39,40]. Normalization and scaling were performed (the ratios and signals were made comparable across slides) using a grid-based Lowess transformation with f = 0.5 (fraction of genes to use); for both channels the intensities of the “Lowess” fraction of genes were added to yield a total signal, and all intensities were divided by this total signal, yielding scaled, arbitrary expression levels.

The microarray results were verified for specific genes by quantitative reverse transcription-PCR using IQt SYBR Green reverse transcription (BioRad) according to the manufacturer’s instructions.

1. Munter S, Way M, Frischknecht F (2006) Signaling during pathogen infection. Sci STKE 2006: re5.

2. Uetz P, Dong YA, Zeretzke C, Atzler C, Baiker A, et al. (2006) Herpesviral protein networks and their interaction with the human proteome. Science 311: 239-242.

3. Stebbins CE (2005) Structural microbiology at the pathogen-host interface. Cell Microbiol 7: 1227-1236.

4. O'Brien KP, Remm M, Sonnhammer EL (2005) Inparanoid: a comprehensive database of eukaryotic orthologs. Nucleic Acids Res 33: D476-480.

5. Diaz-Meco MT, Municio MM, Frutos S, Sanchez P, Lozano J, et al. (1996) The product of par-4, a gene induced during apoptosis, interacts selectively with the atypical isoforms of protein kinase C. Cell 86: 777-786.

6. Sanchez P, De Carcer G, Sandoval IV, Moscat J, Diaz-Meco MT (1998) Localization of atypical protein kinase C isoforms into lysosome-targeted endosomes through interaction with p62. Mol Cell Biol 18: 3069-3080.

7. Kapatral V, Anderson I, Ivanova N, Reznik G, Los T, et al. (2002) Genome sequence and analysis of the oral bacterium Fusobacterium nucleatum strain ATCC 25586. J Bacteriol 184: 2005-2018.

8. Ajdic D, McShan WM, McLaughlin RE, Savic G, Chang J, et al. (2002) Genome sequence of Streptococcus mutans UA159, a cariogenic dental pathogen. Proc Natl Acad Sci U S A 99: 14434-14439.

9. Ventura M, Elli M, Reniero R, Zink R (2001) Molecular microbial analysis of Bifidobacterium isolates from different environments by the species-specific amplified ribosomal DNA restriction analysis (ARDRA). FEMS Microbiol Ecol 36: 113-121.

10. Ryan SM, Fitzgerald GF, van Sinderen D (2006) Screening for and identification of starch-, amylopectin-, and pullulan-degrading activities in bifidobacterial strains. Appl Environ Microbiol 72: 5289-5296.

11. Ewing B, Hillier L, Wendl MC, Green P (1998) Base-calling of automated sequencer traces using phred. I. Accuracy assessment. Genome Res 8: 175-185.

12. Ewing B, Green P (1998) Base-calling of automated sequencer traces using phred. II. Error probabilities. Genome Res 8: 186-194.

13. Staden R, Beal KF, Bonfield JK (2000) The Staden package, 1998. Methods Mol Biol 132: 115-130.

14. Delcher AL, Harmon D, Kasif S, White O, Salzberg SL (1999) Improved microbial gene identification with GLIMMER. Nucleic Acids Res 27: 4636-4641.

15. Schiex T, Gouzy J, Moisan A, de Oliveira Y (2003) FrameD: A flexible program for quality check and gene prediction in prokaryotic genomes and noisy matured eukaryotic sequences. Nucleic Acids Res 31: 3738-3741.

16. Frishman D, Mironov A, Mewes HW, Gelfand M (1998) Combining diverse evidence for gene recognition in completely sequenced bacterial genomes. Nucleic Acids Res 26: 2941-2947.

17. Gish W, States DJ (1993) Identification of protein coding regions by database similarity search. Nat Genet 3: 266-272.

18. Altschul SF, Gish W, Miller W, Myers EW, Lipman DJ (1990) Basic local alignment search tool. J Mol Biol 215: 403-410.

19. Rutherford K, Parkhill J, Crook J, Horsnell T, Rice P, et al. (2000) Artemis: sequence visualization and annotation. Bioinformatics 16: 944-945.

20. Overbeek R, Larsen N, Walunas T, D'Souza M, Pusch G, et al. (2003) The ERGO genome analysis and discovery system. Nucleic Acids Res 31: 164-171.

21. Lowe TM, Eddy SR (1997) tRNAscan-SE: a program for improved detection of transfer RNA genes in genomic sequence. Nucleic Acids Res 25: 955-964.

22. Volfovsky N, Haas BJ, Salzberg SL (2001) A clustering method for repeat analysis in DNA sequences. Genome Biol 2: RESEARCH0027.

23. Henrissat Ca (1999) CAZy database: carbohydrate active enzyme.

24. Busch W, Saier MH, Jr. (2004) The IUBMB-endorsed transporter classification system. Mol Biotechnol 27: 253-262.

25. Gao B, Paramanathan R, Gupta RS (2006) Signature proteins that are distinctive characteristics of Actinobacteria and their subgroups. Antonie Van Leeuwenhoek 90: 69-91.

26. McInerney J, Kooij D (1997) Economic analysis of alternative AD control programmes. Vet Microbiol 55: 113-121.

27. Sonnhammer EL, Durbin R (1995) A dot-matrix program with dynamic threshold control suited for genomic DNA and protein sequence analysis. Gene 167: GC1-10.

28. Rouillard JM, Zuker M, Gulari E (2003) OligoArray 2.0: design of oligonucleotide probes for DNA microarrays using a thermodynamic approach. Nucleic Acids Res 31: 3057-3062.

29. Bilban M, Buehler LK, Head S, Desoye G, Quaranta V (2002) Defining signal thresholds in DNA microarrays: exemplary application for invasive cancer. BMC Genomics 3: 19.

30. Bolstad BM, Irizarry RA, Astrand M, Speed TP (2003) A comparison of normalization methods for high density oligonucleotide array data based on variance and bias. Bioinformatics 19: 185-193.

31. Eisen MB, Spellman PT, Brown PO, Botstein D (1998) Cluster analysis and display of genome-wide expression patterns. Proc Natl Acad Sci U S A 95: 14863-14868.

32. Ventura M, Canchaya C, Del Casale A, Dellaglio F, Neviani E, et al. (2006) Analysis of bifidobacterial evolution using a multilocus approach. Int J Syst Evol Microbiol 56: 2783-2792.

33. Bendtsen JD, Nielsen H, von Heijne G, Brunak S (2004) Improved prediction of signal peptides: SignalP 3.0. J Mol Biol 340: 783-795.

34. Krogh A, Larsson B, von Heijne G, Sonnhammer EL (2001) Predicting transmembrane protein topology with a hidden Markov model: application to complete genomes. J Mol Biol 305: 567-580.

35. Mishra GR, Suresh M, Kumaran K, Kannabiran N, Suresh S, et al. (2006) Human protein reference database--2006 update. Nucleic Acids Res 34: D411-414.

36. Mulder N, Apweiler R (2007) InterPro and InterProScan: tools for protein sequence classification and comparison. Methods Mol Biol 396: 59-70.

37. Ventura M, Zhang Z, Cronin M, Canchaya C, Kenny JG, et al. (2005) The ClgR protein regulates transcription of the clpP operon in Bifidobacterium breve UCC 2003. J Bacteriol 187: 8411-8426.

38. Goffard N, Garcia V, Iragne F, Groppi A, de Daruvar A (2003) IPPRED: server for proteins interactions inference. Bioinformatics 19: 903-904.

39. van Hijum SA, Garcia de la Nava J, Trelles O, Kok J, Kuipers OP (2003) MicroPreP: a cDNA microarray data pre-processing framework. Appl Bioinformatics 2: 241-244.

40. Zomer AL, Buist G, Larsen R, Kok J, Kuipers OP (2007) Time-resolved determination of the CcpA regulon of Lactococcus lactis subsp. cremoris MG1363. J Bacteriol 189: 1366-1381.
